# Association mapping reveals a reciprocal virulence/avirulence locus within diverse US *Pyrenophora teres* f. *maculata* isolates

**DOI:** 10.1186/s12864-022-08529-1

**Published:** 2022-04-09

**Authors:** Shaun J. Clare, Kasia M. Duellman, Jonathan K. Richards, Roshan Sharma Poudel, Lance F. Merrick, Timothy L. Friesen, Robert S. Brueggeman

**Affiliations:** 1 Department of Crop and Soil Sciences, Washington State University, Pullman, WA 99164 USA; 2 Department of Plant, Soil and Entomological Sciences, University of Idaho, Idaho Falls, ID 83402 USA; 3 Department of Plant Pathology and Crop Physiology, Louisiana State University Agricultural Center, Baton Rouge, LA 70808 USA; 4 Department of Plant Pathology, North Dakota State University, Fargo, ND 58108-6050 USA; 5 USDA-ARS, Edward T. Schafer Agricultural Research Center, Cereal Crops Research Unit, Fargo, ND 58102-2765 USA

**Keywords:** *Pyrenophora teres* f. *maculata*, Barley, Association mapping, Reciprocal virulence/avirulence

## Abstract

**Background:**

Spot form net blotch (SFNB) caused by the necrotrophic fungal pathogen *Pyrenophora teres* f. *maculata* (*Ptm*) is an economically important disease of barley that also infects wheat. Using genetic analysis to characterize loci in *Ptm* genomes associated with virulence or avirulence is an important step to identify pathogen effectors that determine compatible (virulent) or incompatible (avirulent) interactions with cereal hosts. Association mapping (AM) is a powerful tool for detecting virulence loci utilizing phenotyping and genotyping data generated for natural populations of plant pathogenic fungi.

**Results:**

Restriction-site associated DNA genotyping-by-sequencing (RAD-GBS) was used to generate 4,836 single nucleotide polymorphism (SNP) markers for a natural population of 103 *Ptm* isolates collected from Idaho, Montana and North Dakota. Association mapping analyses were performed utilizing the genotyping and infection type data generated for each isolate when challenged on barley seedlings of thirty SFNB differential barley lines. A total of 39 marker trait associations (MTAs) were detected across the 20 barley lines corresponding to 30 quantitative trait loci (QTL); 26 novel QTL and four that were previously mapped in *Ptm* biparental populations. These results using diverse US isolates and barley lines showed numerous barley-*Ptm* genetic interactions with seven of the 30 *Ptm* virulence/avirulence loci falling on chromosome 3, suggesting that it is a reservoir of diverse virulence effectors. One of the loci exhibited reciprocal virulence/avirulence with one haplotype predominantly present in isolates collected from Idaho increasing virulence on barley line MXB468 and the alternative haplotype predominantly present in isolates collected from North Dakota and Montana increasing virulence on barley line CI9819.

**Conclusions:**

Association mapping provided novel insight into the host pathogen genetic interactions occurring in the barley-*Ptm* pathosystem. The analysis suggests that chromosome 3 of *Ptm* serves as an effector reservoir in concordance with previous reports for *Pyrenophora teres* f. *teres*, the causal agent of the closely related disease net form net blotch*.* Additionally, these analyses identified the first reported case of a reciprocal pathogen virulence locus. However, further investigation of the pathosystem is required to determine if multiple genes or alleles of the same gene are responsible for this genetic phenomenon.

**Supplementary Information:**

The online version contains supplementary material available at 10.1186/s12864-022-08529-1.

## Background

*Pyrenophora teres* f. *maculata* (*Ptm*) is a globally important necrotrophic foliar pathogen that causes the disease spot form net blotch (SFNB) of barley. In some growing regions, SFNB is considered the most prevalent foliar disease of barley [1] with severity reported up to 55% on the upper leaves [2] corresponding to yield losses of 44%. In recent years, *Ptm* has also been identified in the field infecting wheat [3, 4], which raises the alarm of an emerging wheat pathogen that could potentially cause yield and quality losses in this globally important crop.

*Pyrenophora teres* f. *maculata* and its close relative, *Pyrenophora teres* f. *teres* (*Ptt*) the cause of net form net blotch [5, 6], produce at least four toxins, known as Toxins A, B, C and D that are secondary metabolites produced in the same biosynthetic pathway [7–10]. However, many of the effectors utilized by necrotrophic pathogens have been shown to be proteinaceous effectors that interact with and activate host immunity receptor responses [11]. These host immunity receptors typically induce programmed cell death (PCD) that evolved to provide resistance against biotrophic pathogens that require living host cells to extract nutrients from the host. In these typical gene-for-gene resistance interactions, the host immune system recognizes the presence of the biotrophic pathogen, sequesters it and its feeding structures within foci of dead cells, restricting access to nutrients and effectively stops colonization. However, necrotrophic pathogens that can acquire nutrients from dying and dead tissue evolved necrotrophic effectors (NEs) that are recognized by host immunity receptors eliciting PCD immunity responses. These inverse gene-for-gene interactions resulting from necrotrophic effector triggered susceptibility (NETS) [12] have been characterized for other members of the Dothideomycetes [11]. Thus, diverse necrotrophic fungal pathogens have evolved to hijack the major plant immunity mechanisms to proliferate, and complete their lifecycles utilizing NETS to facilitate disease development [12]. This contrasts with traditional gene-for-gene relationships characterized in plant-biotrophic pathogen incompatible interactions [13]. Interestingly, *Ptm* undergoes an early latent phase during colonization, where structures resembling haustoria form indicating a brief biotrophic phase, followed by the rapid conversion to a necrotrophic lifestyle [14]. Considering that *Ptm* may be a hemi-biotroph [6], both gene-for-gene and inverse gene-for-gene host-parasite genetic interactions determining avirulence or virulence could occur in the barley*-Ptm* pathosystem.

Genetic mapping utilizing *Ptt* bi-parental populations have identified marker trait associations (MTA) with avirulence that follow the gene-for-gene model [15–17]. However, NEs hypothesized to function in inverse gene-for-gene interactions have been predominantly mapped using *Ptt* bi-parental populations [15–19] and more recently utilizing association mapping (AM) [20]. Only a single study has reported on the use of a *Ptm* bi-parental population to map virulence loci [21] and to date AM utilizing a natural *Ptm* population had not been reported. Avirulence and virulence effectors produced by *Ptm* and *Ptt* have been shown to be unique based on genetic analyses and it was hypothesized that host resistance genes for the two pathogens only showed partial overlap [5] suggesting that the two pathogens should be considered distinct when deploying host resistance genes.

Two-enzyme restriction-site association DNA genotyping-by-sequencing (RAD-GBS) has been shown as effective method to genotype *P. teres* and performed exclusively in bi-parental populations [19, 21, 22]. AM is a powerful alternate approach to bi-parental population development that has been successfully applied in the barley-*P. teres* pathosystem to identify markers associated with host resistance/susceptibility loci [23–33] and recently pathogen avirulence/virulence loci within *Ptt* [20]. The first *Ptt* AM study identified 14 unique genomic loci associated with virulence, with four of the loci validated by quantitative trait loci (QTL) mapping in two bi-parental populations [20]. In the past decade, optimization of AM algorithms have been achieved with the most recent association mapping package released being Bayesian-information and Linkage-disequilibrium Iteratively Nested Keyway (BLINK) in 2018 [34]. The BLINK package utilizes linkage disequilibrium (LD) information to infer the relatedness of individuals to replace the previous binning method of SUPER [35] and FarmCPU [36] algorithms. The BLINK algorithms effectively reduced the number of steps required by the user (LD pruning and kinship construction), and computational burden, while simultaneously suppressing false positive and increasing true positive MTAs [34].

For the first time we report on the use of AM to identify MTA with virulence or avirulence loci utilizing a population of *Ptm*. Thirty differential barley lines [37] were used to phenotype a population of 103 *Ptm* isolates collected from the three highest producing barley states of the United States (Table 1). This work provides the initial genetic information to identify and characterize NEs or other genetic factors that determine *Ptm* virulence or avirulence*.* The effectors underlying these loci determine the outcome, compatibility (susceptibility) -vs- incompatibility (resistance), in this complex pathosystem and the information generated will aid in the intelligent deployment of durable resistance. In the barley-*P. teres* pathosystem, it has become apparent that breeding strategies will require the elimination of host susceptibility targets that function within the inverse gene-for-gene model while maintaining resistance mechanisms that function in the classical gene-for-gene mechanism. Adding to this complexity are important loci with antagonistic host resistance and susceptibility genes that are genetically linked within loci [5].

**Table 1:** Collection information of *Pyrenophora teres* f. *maculata* isolate populations used in this study

				Isolates	
State	Location(s)	Sampling Date	Cultivar^a^	Collected	Assessed
North Dakota	Fargo, Langdon, Dickinson, Nesson Valley	Jun/Jul 2012	Pinn/Trad	91	68
Montana	Blackfoot	Jun 2012	Pinn/Trad	49	15
Idaho	Sidney	Jun 2013	Mor 69	42	20
			Total	182	103

^a^Pinn/Trad are the barley cultivars Pinnacle and Tradition, Mor 69 is the barley cultivar Moravian 69

## Results

### Association mapping panel

Genotyping data that met quality parameters such as optimization for high quality SNPs, filtering for < 35% missing data, ≥ 3 minimum read depth, and 2 maximum alleles, yielded 4,836 informative SNPs spread across the twelve *Ptm* chromosomes for 103 of the 177 isolates evaluated for virulence. Thus, 103 of the *Ptm* isolates were sufficiently genotyped and were included in the AM panel.

**Fig. 1:**
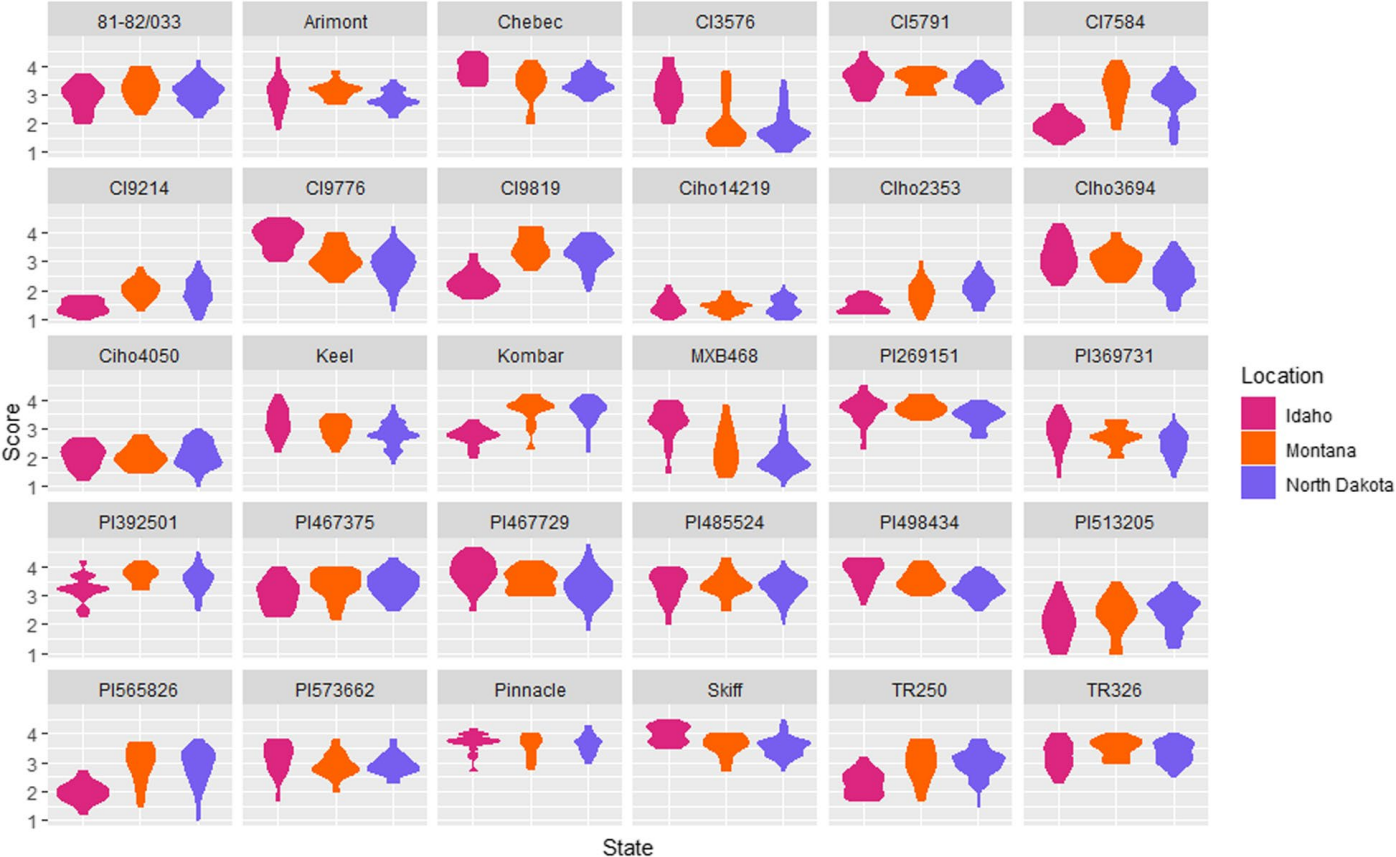
Violin plot showing the phenotypic distribution of *Pyrenophora teres* f. *maculata* subpopulations across barley lines and grouped by location. Generated using ggplot2 3.3.2 [38] in *R 3.6.3*

The phenotypic distributions of the 103 genotyped isolates varied on the 30 barley lines challenged with the *Ptm* isolates (Table 2, Fig. 1). Five barley lines CI3576, CI9776, CIho3694, MXB468, and PI467729 showed a strong differential response of 3 or more, with infection types ranging from 1.0 to 4.3 (2.87 ± 0.80), 1.3 to 4.5 (2.46 ± 0.65), 1.3 to 4.3 (2.31 ± 0.60), 1.3 to 4.3 (2.19 ± 0.74), and 1.8 to 4.8 (3.38 ± 0.55, Table 2), respectively. Eighteen barley lines showed a moderate differential response of 2 to less than 3 (Table 2). Seven of the thirty barley lines showed poor differential lesion reactions, with differences between the most and least virulent isolates being 1.8 or less on Pinnacle, Ciho14219, Skiff, CI5791, PI485524, PI498434, and TR326 (Table 2).

**Table 2:** The phenotypic distribution of isolates inoculated on barley lines on which significant marker trait associations were identified plus controls. The arrow next to the range indicates the phenotypic differential from high (up arrow), medium (up-down arrow) and low (down arrow)

	*Ptm* Scale						
Genotype	Min	Max	Range	Avirulent	Virulent	Best Model	MTA
Pinnacle^a^	2.7	4.3	1.6 ↓	2	101	BLINK_PC4_	-
CIho14219^b^	1.0	2.2	1.2 ↓	103	0	BLINK_PC4_	-
81–82/033	2.0	4.2	2.2 ↕	36	67	BLINK_PC4+Binary_	-
Arimont	1.8	4.3	2.5 ↕	56	47	BLINK_PC4+Binary_	-
Chebec	2.0	4.5	2.5 ↕	5	98	BLINK_PC15+Binary_	4
Keel	1.8	4.2	2.4 ↕	53	50	BLINK_PC4_	-
Kombar	2.0	4.2	2.2 ↕	22	81	BLINK_Binary_	4
Skiff	2.7	4.5	1.8 ↓	2	101	BLINK_PC4_	-
CI3576	1.0	4.3	3.3 ↑	83	20	BLINK	2
CI5791	2.7	4.5	1.8 ↓	3	100	BLINK_PC15+Binary_	1
CI7584	1.3	4.5	2.9 ↕	50	53	BLINK_Binary_	1
CI9214	1.0	3.0	2.0 ↓	101	2	BLINK	2
CI9776	1.3	4.5	3.2 ↑	40	63	BLINK_Binary_	1
CI9819	1.7	4.2	2.5 ↕	33	70	BLINK_Binary_	1
CIho2353	1.0	3.0	2.0 ↕	101	2	BLINK_PC15_	-
CIho3694	1.3	4.3	3.0 ↑	64	39	BLINK	2
CIho4050	1.0	3.0	2.0 ↕	102	1	BLINK_PC15_	3
MXB468	1.0	4.0	3.0 ↑	79	24	BLINK_PC4_	1
PI269151	2.3	4.5	2.2 ↕	10	93	BLINK_PC4+Binary_	1
PI369731	1.3	3.8	2.5 ↕	78	23	BLINK	1
PI392501	2.3	4.5	2.2 ↕	9	94	BLINK_PC4_	-
PI467375	2.2	4.3	2.1 ↕	20	83	BLINK_Binary_	1
PI467729	1.8	4.8	3.0 ↑	15	88	BLINK	2
PI485524	2.0	4.3	2.3 ↕	16	87	BLINK_PC4+Binary_	-
PI498434	2.5	4.3	1.8 ↓	10	93	BLINK_PC4+Binary_	2
PI513205	1.0	3.5	2.5 ↕	86	17	BLINK_PC4_	-
PI565826	1.0	3.8	2.8 ↕	58	45	BLINK_PC15_	1
PI573662	1.7	3.8	2.1 ↕	50	53	BLINK	4
TR250	1.5	3.8	2.3 ↕	52	51	BLINK	3
TR326	2.3	4.0	1.7 ↓	12	91	BLINK_PC4+Binary_	2

^a^Susceptible check

^b^Resistant check

### Linkage disequilibrium, population structure and kinship

Four principal components, Q4, explained 26.3% of the variation, and fifteen principal components, Q15, explained 50.2% of the variation (Supplemental Fig. 1 & 2). Both were used as cofactors in the mixed model analyses. The first four principal components explained 11.8%, 7.8%, 3.7% and 2.9% of the variation, with the remaining eleven principal components accounting for 1.8–2.6% of the variation (Supplemental Fig. 2). As STRUCTURE analysis only revealed two subpopulations despite the use of six sampling locations (Supplemental Fig. 3), this was not used as a covariate as these would be linearly correlated. The eastern North Dakota (ND) isolates were more closely related to the Montana (MT) isolates, with western ND isolates forming a separate population despite being in closer proximity to MT. The Idaho (ID) isolates formed an admixture population of the two subpopulations. In addition, construction of an EMMA kinship matrix corroborated these results with western ND forming two clusters, eastern ND and MT forming two clusters and ID forming an admixture group (data not shown).

Linkage disequilibrium decay was estimated by performing local polynomial regression of R^2^ values from pairwise comparisons of all markers using physical distances along the chromosome (Supplemental File 1). The genome wide LD decay was estimated to be approximately 7 kbp at an R^2^ of 0.1, with rapid decay from half of the maximum at approximately 4.2 kbp to background level at approximately 75 kbp (Supplemental Fig. 4 & 5).

### Association mapping analyses

To control for false positives, two naïve models and six different mixed models (BLINK, BLINK_Binary_, BLINK_PC4_, BLINK_PC4+Binary,_ BLINK_PC15,_ and BLINK_PC15+Binary_) were evaluated to identify MTAs for *Ptm* virulence/avirulence using the 1–5 *Ptm* infection type scale and binary scale (virulent or avirulent) and for the mating type (MAT) locus as an additional control. Because LD is used to construct the kinship matrix in BLINK, neither LD pruning, nor a kinship matrix are required [34]. The current factors that determine the number of models utilized is dependent on accounting for population structure and ensuring that the phenotype scale has sufficient differential power. To address this, we corrected for population structure by using principal components accounting for 25 and 50% of the genetic variation and used both a standard phenotyping scale [37] and a binary conversion to increase the differential power. The model with the best fit to the expected *p-*values (QQ plot) and lowest mean square difference (MSD) (Table 2; Supplemental Table 2) was selected as optimal.

**Fig. 2:**
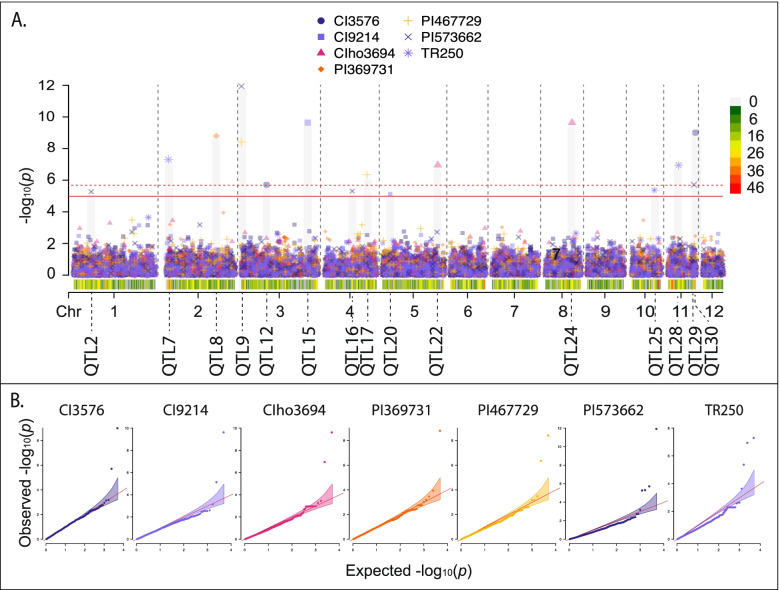
**A** Manhattan plot for barley lines where the standard BLINK model utilizing the 1–5 *Ptm* phenotyping scale was identified as the optimal model. Bonferroni correction threshold is indicated by the solid (α-level 0.05) and dashed (α-level 0.01) red lines. SNP density is indicated along the bottom of the plot with the corresponding heat scale shown to the left and the 12 *Pyrenophora teres* f. *maculata* chromosomes (Chr) designated below. The QTL designations are given below each chromosome. **B** QQ plots for corresponding lines within the standard BLINK model Manhattan plot with 95% confidence interval shown by the shaded color. The Manhattan and QQ plots were generated using CMplot [39] in *R 3.6.3*

**Fig. 3:**
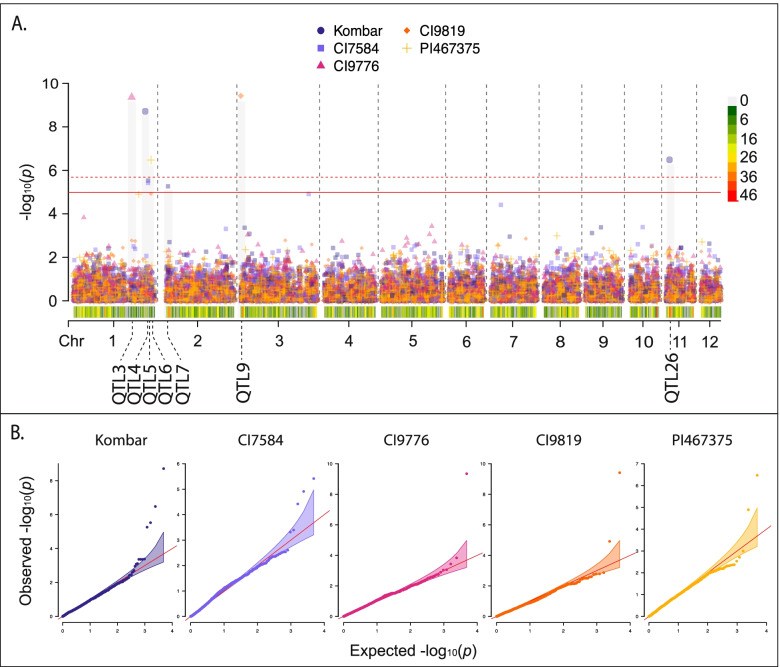
**A** Manhattan plot for barley lines where the binary BLINK model was the identified as the optimal model. Bonferroni correction threshold is indicated by the solid (α-level 0.05) and dashed (α-level 0.01) red lines. SNP density is indicated along the bottom of the plot with the corresponding heat scale shown to the left and the 12 *Pyrenophora teres* f. *maculata* chromosomes (Chr) designated below. The QTL designations are given below each chromosome. **B** QQ plots for corresponding lines within the binary BLINK model Manhattan plot with 95% confidence interval shown by the shaded color. The Manhattan and QQ plots were generated using CMplot [39] in *R 3.6.3*

**Fig. 4:**
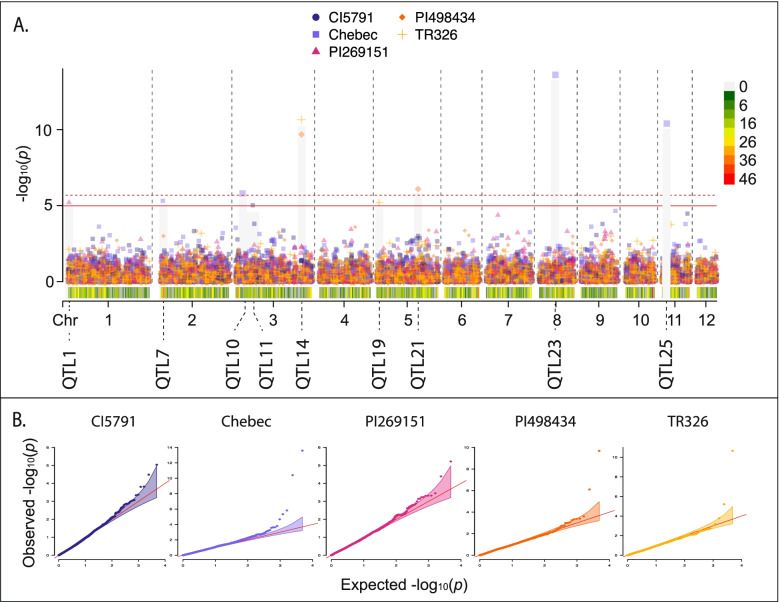
**A** Manhattan plot for barley lines where the binary BLINK model accounting for population structure (PC4 or PC15) was identified as the optimal model. Bonferroni correction threshold is indicated by the solid (α-level 0.05) and dashed (α-level 0.01) red lines. SNP density is indicated along the bottom of the plot with the corresponding heat scale shown to the left and the 12 *Pyrenophora teres* f. *maculata* chromosomes (Chr) designated below. The QTL designations are given below each chromosome. **B** QQ plots for corresponding lines within the binary BLINK account for population structure model Manhattan plot with 95% confidence interval shown by the shaded color. The Manhattan and QQ plots were generated using CMplot [39] in *R 3.6.3*

**Fig. 5:**
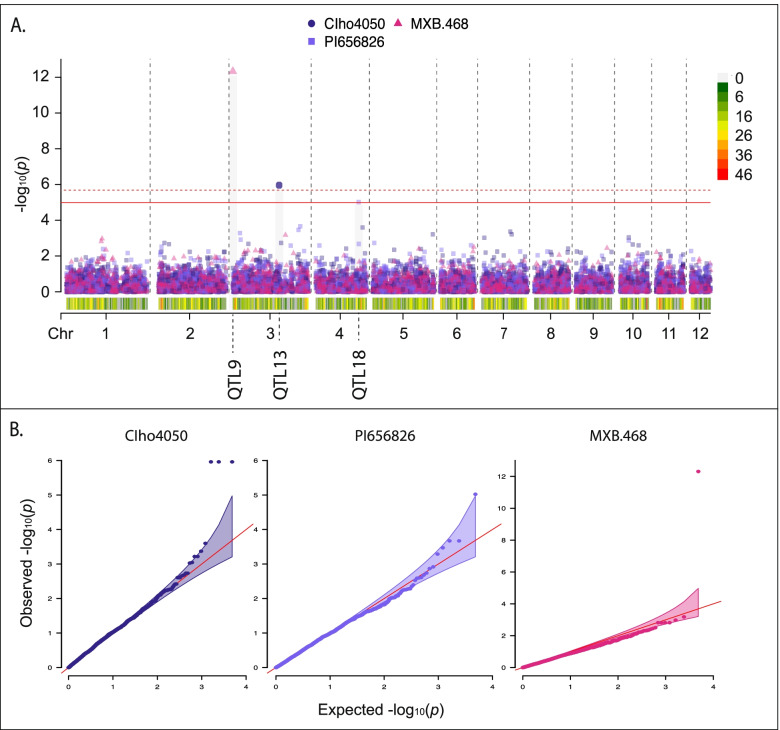
**A** Manhattan plot for barley lines where the standard BLINK model utilizing the 1–5 Ptm scale phenotyping and accounting for population structure (PC4 or PC15) was identified as the optimal model. Bonferroni correction threshold is indicated by the solid (α-level 0.05) and dashed (α-level 0.01) red lines. SNP density is indicated along the bottom of the plot with the corresponding heat scale shown to the left and the 12 *Pyrenophora teres* f. *maculata* chromosomes (Chr) designated below. The QTL designations are given below each chromosome. **B** QQ plots for corresponding lines within the binary BLINK model Manhattan plot with 95% confidence interval shown by the shaded color. The Manhattan and QQ plots were generated using CMplot [39] in *R 3.6.3*

No single model was best for all interactions; however, the standard BLINK model was the optimal model with seven of the 30 barley lines identifying significant MTAs (Fig. 2), followed by five in the binary BLINK models (Fig. 3), three in the binary BLINK model with population structure (PC4, Fig. 4), two in both the binary (Fig. 4) and standard (Fig. 5) models incorporating additional population structure (PC15) and one in the standard BLINK model incorporating population structure (PC4, Fig. 5). The standard and binary BLINK models accounting for additional population structure (PC15) generally overfitted the models, except in the case of CIho4050 and PI565826 in the standard model and Chebec and CI5791 in the binary model. Therefore, these models were visualized together with the standard and binary PC4 models. The MAT type was used as the phenotypic control, and all models successfully identified a significant MTA 6.5 kb proximal to the known MAT type locus on *Ptm* chromosome 9 (Supplemental Fig. 6).

Twenty out of the thirty barley genotype-*Ptm* interactions yielded 39 significant MTAs for virulence/avirulence across eight of the twelve chromosomes, corresponding to 30 unique loci (Figs. 2, 3, 4 and 5, Table 3). All five of the barley lines showing strong differential phenotypic scores (≥ 3) identified significant MTAs. Also, eleven out of the 18 barley lines showing moderate differential responses (< 3.0 > 1.8) and four out of seven barley lines showing poor phenotypic differential reactions (≤ 1.8) identified significant MTAs in the analyses. The majority of the barley lines identified one significant MTA (nine lines), followed by two (six lines), four (three lines) and three MTAs (two lines, Tables 2 and 3). MTAs were not identified on the resistant (CIho14219) and susceptible (Pinnacle) checks, along with 81–82/033, Arimont, Keel, Skiff, CIho2353, PI392501, PI485524, and PI513205. In addition, the standard BLINK model, binary BLINK model, binary models accounting for population structure (PC4 and PC15), and standard BLINK with population structure (PC4 and PC15) identified 16, eight, five, five, one and four significant MTAs, respectively (Table 2).

**Table 3:** Significant marker trait associations identified across all models and barley lines

**QTL**	**Marker**	**Chr**	**Pos**	**Genotype**	**Model**	**LOD**	**Sig. Thres**	**MAF**	**Effect** ± **SE**	***R***^*2*^	**Scale**	**Inferred Locus**	**Genotypes from Inferred Locus**
*Ptm_QTL1*	1_153947	1	153,947	PI269151	BLINK_PC4+Binary_	5.22	0.05	0.07	-0.38 ± 0.08	19.70%	Binary	*vQTL1ABC*	Skiff, TR326, 81–82/033, PI392501
*Ptm_QTL2*	1_1198910	1	1,198,910	PI573662	BLINK	5.26	0.05	0.10	-0.29 ± 0.09	8.70%	Full		
*Ptm_QTL3*	1_3793576	1	3,793,576	CI9776	BLINK_Binary_	9.35	0.01	0.13	-0.53 ± 0.10	22.70%	Binary		
*Ptm_QTL4*	1_4658734	1	4,658,734	Kombar	BLINK_Binary_	8.71	0.01	0.02	-0.83 ± 0.17	18.80%	Binary	*QTL1ABC*	Innovation, Hockett, Lacey, Pinnacle, Quest, Stellar
*Ptm_QTL5*	1_4837851	1	4,837,851	Kombar	BLINK_Binary_	5.53	0.05	0.10	-0.48 ± 0.09	23.00%	Binary		
	1_4837851	1	4,837,851	CI7584	BLINK_Binary_	5.41	0.05	0.10	-0.65 ± 0.10	28.80%	Binary		
*Ptm_QTL6*	1_5027390	1	5,027,390	PI467375	BLINK_Binary_	6.47	0.01	0.14	-0.38 ± 0.08	19.50%	Binary		
*Ptm_QTL7*	2_474724	2	474,724	TR250	BLINK	7.29	0.05	0.08	-0.68 ± 0.13	22.70%	Full		
	2_474724	2	474,724	Kombar	BLINK_Binary_	5.62	0.05	0.08	-0.55 ± 0.09	25.90%	Binary		
	2_481844	2	481,844	Chebec	BLINK_PC15+Binary_	5.33	0.05	0.06	0.05 ± 0.06	0.70%	Binary		
*Ptm_QTL8*	2_3522060	2	3,522,060	PI369731	BLINK	8.76	0.01	0.08	0.68 ± 0.12	25.20%	Full		
*Ptm_QTL9*	3_63095	3	63,095	PI467729	BLINK	8.4	0.01	0.14	0.59 ± 0.11	22.20%	Full		
	3_63095	3	63,095	CI9819	BLINK_Binary_	9.41	0.01	0.14	-0.63 ± 0.08	37.60%	Binary		
	3_63095	3	63,095	MXB468	BLINK_PC4_	12.3	0.01	0.14	1.22 ± 0.11	57.00%	Full		
	3_63095	3	63,095	PI573662	BLINK	11.9	0.01	0.14	0.43 ± 0.07	24.10%	Full		
*Ptm_QTL10*	3_494752	3	494,752	Chebec	BLINK_PC15+Binary_	5.82	0.01	0.03	-0.56 ± 0.06	43.20%	Binary		
*Ptm_QTL11*	3_1137083	3	1,137,083	CI5791	BLINK_PC15+Binary_	5.07	0.05	0.10	-0.08 ± 0.04	3.70%	Binary		
*Ptm_QTL12*	3_1659917	3	1,659,917	CI3576	BLINK	5.7	0.01	0.08	1.32 ± 0.17	37.60%	Full		
*Ptm_QTL13*	3_3029308	3	3,029,308	CIho4050	BLINK_PC4_	5.01	0.05	0.11	-0.36 ± 0.09	13.50%	Full		
	3_3029419	3	3,029,419	CIho4050	BLINK_PC4_	5.01	0.05	0.11	-0.36 ± 0.09	13.50%	Full		
	3_3029420	3	3,029,420	CIho4050	BLINK_PC4_	5.01	0.05	0.11	-0.36 ± 0.09	13.50%	Full		
*Ptm_QTL14*	3_4245269	3	4,245,269	TR326	BLINK_PC4+Binary_	10.66	0.01	0.02	-0.58 ± 0.12	18.20%	Binary	*QTL3B*	Stellar, Lacey
	3_4247579	3	4,247,579	PI498434	BLINK_PC4+Binary_	9.69	0.01	0.03	-0.61 ± 0.11	22.90%	Binary	*QTL3B*	Stellar, Lacey
*Ptm_QTL15*	3_4268498	3	4,268,498	CI9214	BLINK	9.62	0.01	0.19	-0.42 ± 0.08	20.20%	Full	*QTL3B*	Stellar, Lacey
*Ptm_QTL16*	4_1832576	4	1,832,576	PI573662	BLINK	5.31	0.05	0.03	0.18 ± 0.14	1.40%	Full		
*Ptm_QTL17*	4_2772570	4	2,772,570	PI467729	BLINK	6.34	0.01	0.03	0.79 ± 0.20	13.10%	Full		
*Ptm_QTL18*	4_2863635	4	2,863,635	PI565826	BLINK_PC15_	5.02	0.05	0.02	-0.17 ± 0.31	0.30%	Full		
*Ptm_QTL19*	5_186459	5	186,459	TR326	BLINK_PC4+Binary_	5.21	0.05	0.03	-0.34 ± 0.12	6.90%	Binary		
*Ptm_QTL20*	5_516326	5	516,326	CI9214	BLINK	5.13	0.05	0.07	0.31 ± 0.13	5.60%	Full		
*Ptm_QTL21*	5_2662282	5	2,662,282	PI498434	BLINK_PC4+Binary_	6.11	0.01	0.01	-0.42 ± 0.15	7.50%	Binary	*vQTL3*	Skiff
*Ptm_QTL22*	5_3532773	5	3,532,773	CIho3694	BLINK	6.95	0.01	0.07	-0.57 ± 0.16	11.60%	Full		
*Ptm_QTL23*	8_1149348	8	1,149,348	Chebec	BLINK_PC15+Binary_	13.6	0.01	0.03	-0.10 ± 0.08	1.40%	Binary		
*Ptm_QTL24*	8_1808397	8	1,808,397	CIho3694	BLINK	9.62	0.01	0.09	0.82 ± 0.11	27.90%	Full		
*Ptm_QTL25*	10_1598365	10	1,598,365	TR250	BLINK	5.37	0.05	0.31	0.07 ± 0.11	0.40%	Full		
*Ptm_QTL26*	11_309227	11	309,227	Kombar	BLINK_Binary_	6.49	0.01	0.03	0.23 ± 0.16	2.00%	Binary		
*Ptm_QTL27*	11_393533	11	393,533	Chebec	BLINK_PC15+Binary_	10.41	0.01	0.03	-0.41 ± 0.07	22.80%	Binary		
*Ptm_QTL28*	11_760041	11	760,041	TR250	BLINK	6.94	0.01	0.06	0.67 ± 0.15	16.50%	Full		
*Ptm_QTL29*	11_1735066	11	1,735,066	PI573662	BLINK	5.71	0.01	0.02	-0.47 ± 0.17	6.50%	Full		
*Ptm_QTL30*	11_1834542	11	1,834,542	CI3576	BLINK	8.99	0.01	0.17	1.14 ± 0.12	45.40%	Full		
	1_4243533	1	4,243,533	PI467375	BLINK_Binary_	4.89	N/A	0.05	-0.53 ± 0.11	18.80%	Binary		
*Ptm_QTL6*	1_5027390	1	5,027,390	CI9819	BLINK_Binary_	4.92	N/A	0.14	-0.54 ± 0.09	27.20%	Binary		
	3_4383898	3	4,383,898	CI7584	BLINK_Binary_	4.91	N/A	0.26	0.49 ± 0.09	24.50%	Binary		
	6_1258294	6	1,258,294	Keel	BLINK_PC4_	4.93	N/A	0.14	0.32 ± 0.09	10.20%	Full		

In respect to chromosomes, significant MTAs were detected on chromosome 1 (seven MTAs), chromosome 2 (four MTAs), chromosome 3 (13 MTAs), chromosome 4 (three MTAs), chromosome 5 (four MTAs), chromosome 8 (two MTAs), chromosome 10 (one MTA), and chromosome 11 (five MTAs). MTAs were clustered together into a quantitative trait locus (QTL) if they were within 7 kbp based on the LD decay estimates to be 7 kbp at an *R*^*2*^ value of 0.1. Based on the clustering for QTL designations, QTL *Ptm_QTL9* was identified with four barley lines (CI9819, MXB468, PI467729 and PI573662), exhibiting reciprocal virulence in that both haplotypes provide virulence on differing barley lines, QTL *Ptm_QTL7* with three lines (Chebec, Kombar and TR250), and QTL *Ptm_QTL5* (Kombar and CI7584) and *Ptm_QTL14* (TR326 and PI498434) with two lines. Despite no models identifying a significant MTA for the barley line Keel, one that corresponded to a novel QTL on chromosome 6 was nearly significant (Table 3).

The lines CI3576 and CI9776 show a similar phenotypic distribution as MXB468 with increased susceptibility to the ID isolates. Likewise, the lines CI7584, CI9214, Kombar, and TR250 show similar phenotypic distribution to CI9819 with increased susceptibility to the ND and MT isolates. However, in these cases the reciprocal virulence locus (*Ptm_QTL9*) was not identified as being responsible. Instead, for CI3576 two QTL designated *Ptm_QTL12* and *Ptm_QTL30* were identified on chromosomes 3 and 11 accounting for 37.6 and 45.4% of the phenotypic variation, respectively. In the case of CI9776, the locus *Ptm_QTL3* was identified on chromosome 1 and accounted for 22.7% of the variation. In the case of CI7584, one locus designated *Ptm_QTL5,* was also identified on chromosome 1 accounting for 28.8% of the phenotypic variation. For CI9214, two loci were identified with *Ptm_QTL15* on chromosome 3 and *Ptm_QTL20* on chromosome 5 accounting for 20.2 and 5.6% of the phenotypic variation. On Kombar, four QTL were detected, *Ptm_QTL4, Ptm_QTL5, Ptm_QTL7,* and *Ptm_QTL26* on chromosomes 1, 1, 2 and 11 that account for 18.8, 23.0, 25.9 and 2.0% of the phenotypic variation. *Ptm_QTL4* has previously been mapped as *QTL1ABC* in a P-A14/CAWB05Pt-4 biparental mapping population (pers. comm. Tim Friesen). For the TR250 barley line, a total of three QTL were identified, *Ptm_QTL7, Ptm_QTL25* and *Ptm_QTL28* on chromosomes 2, 10 and 11 accounting for 22.7, 0.4 and 16.5%, respectively. In respect to Chebec, a further three QTL were identified in addition to *Ptm_QTL7* that only accounted for 0.7% of the phenotypic variation*.* The remaining QTL identified with Chebec include *Ptm_QTL10, Ptm_QTL23* and *Ptm_QTL27* on chromosomes 3, 8 and 11 that account for 43.2, 1.4 and 22.8% of the phenotypic variation.

A total of 202 candidate genes were identified within the 30 unique loci (Supplemental File 2), ranging from one to 23 candidate genes. Of the 202 translated proteins, a total of ten and 78 are predicted to be apoplastic or cytoplasmic effectors, respectively (Supplemental Table 3). This resulted in a total of 85 and 117 predicted effectors and non-effectors, respectively, as EffectorP 3.0 occasionally is not conclusive in determining if a predicted effector is apoplastic or cytoplasmic. Of the predicted non-effector translated proteins, these proteins contain homology to proteins such as transporters, catalytic enzymes, secondary metabolite synthesis enzymes and inhibitors.

## Discussion

The *Ptm* isolates used in this study were collected from the three highest barley producing states within the US [40] spread across the Pacific Northwest and the Upper Midwestern US representing a large geographic region (1240 km from Blackfoot, ID to Langdon, ND). The Pacific Northwest and Upper Midwest are separated by the Rocky Mountains and may be the cause of the two subpopulations identified via EMMA kindship matrix and STRUCTURE analysis. AM algorithms have seen iterative improvements over the years to the most recent release of BLINK [34], that provides numerous benefits in usability, processing time and model reduction. This allowed for the detection of considerable amount of significant MTAs identified in this study despite the stringent cutoff. When assessing the gene space of each QTL, several QTL only contained a single candidate gene indicating the power of BLINK (Supplemental Table 3). In addition, all QTL either have predicted effectors or genes predicted to have homology to genes that facilitate pathogenicity such as transporters for nutrient acquisition [41], secondary metabolite synthesis enzymes to attack the host [42], and inhibitors and catalytic enzymes for protection [43].

Barley lines of particular interest that failed to identify significant MTA were 81–82/033, Skiff and PI392501. These lines have previously been used to map association with virulence in bi-parental populations between the US isolate FGOB10Ptm-1 and the Australian isolate SG1 [21]. Carlsen et al. [21] identified two to four QTL with 81–82/033, Skiff, PI392501 and TR326. This would suggest that effectors interacting with 81–82/033, Skiff and PI392501 are fixed or at low frequency within the US population compared to the wider cross of FGOB10Ptm-1 and SG1 used by Carlsen et al. [21] and therefore were not detected in this study. The fourth line used by Carlsen et al. [21], TR326 had previously identified two QTL, *vQTL1A* and *vQTL5*, on chromosomes 1 and 5, respectively, with both being contributed by FGOB10Ptm-1 and not identified in this study. However, the two QTL *Ptm_QTL14* and *Ptm_QTL19* identified in this study present on chromosomes 3 and 5, respectively, were not identified in the previous bi-parental mapping study. The bi-parental and association mapping both identified a QTL on chromosome 5 using TR326, however, these loci did not colocalize and thus appear to be distinct. In addition, the locus *vQTL1ABC* identified with all four lines by Carlsen et al. [21] was also identified with PI269151 in this study suggesting this region may harbor multiple virulence/avirulence effectors. The locus *Ptm_QTL14,* identified with TR326 and PI498434 colocalizes with *QTL3B* identified in a biparental mapping population of US isolate P-A14 and US wild barley grass isolate CAWB05Pt-4 (Friesen, T. et al. unpublished). Based on the interval of *QTL3B* this also places *Ptm_QTL15* under the same locus. The fact that two loci were identified via association mapping and one single broader QTL in biparental mapping may be due to the use of a natural population that has undergone more recombination to break the linkage between these two loci.

**Fig. 6:**
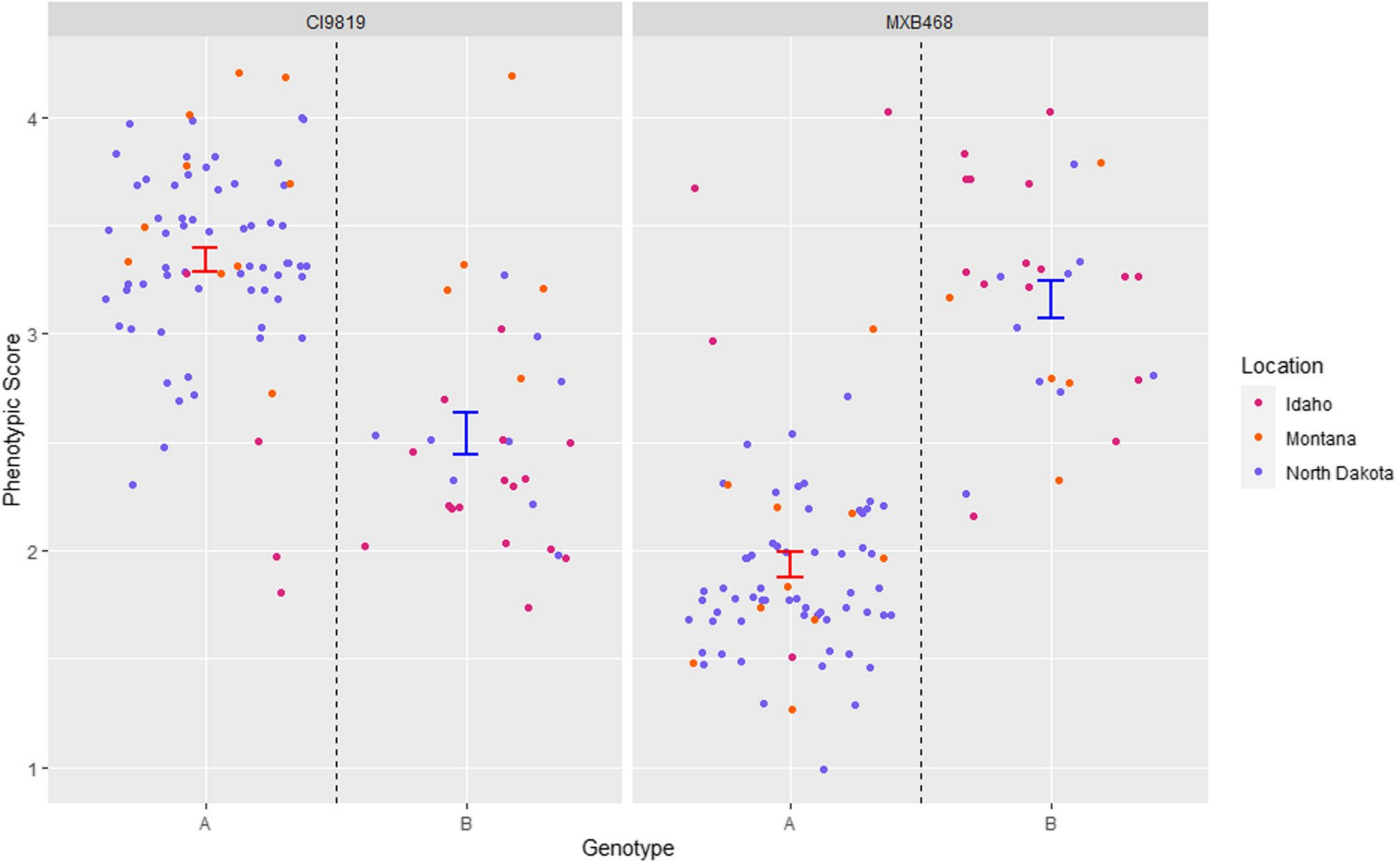
Jitter genotype by phenotype plot for alleles A and B of the marker 3_630965 on barley line (**A**) MXB468 and (**B**) CI9819 using the 1–5 *Ptm* phenotyping scale [37] identified as the potential reciprocal virulence locus. Phenotypic scores are color coded based on isolate origin and standard error is displayed in blue for allele A and red for allele B

The most interesting locus, *Ptm_QTL9,* was identified with CI9819, MXB468, PI467729, PI573662 (Figs. 2, 3 and 5). The barley line CI9819 exhibited increased susceptibility to the ND and MT subpopulation isolates, whereas MXB468, PI467729 and PI573662 exhibited increased susceptibility to the ID isolates (Fig. 1). *Ptm_QTL9* was the only QTL identified on barley lines CI9819 and MXB468 whereas, *Ptm_QTL17* was identified with barley line PI467729 and *Ptm_QTL2, Ptm_QTL16* and *Ptm_QTL29* with barley line PI573662. However, *Ptm_QTL9* was still the major locus identified with PI467729 and PI573662. To date, *Ptm* and *Ptt* effectors have shown virulence/avirulence dichotomy [5], with the previous case of reciprocal virulence profiles mapping to separate loci in the *Ptt* bi-parental cross between isolates 15A and 6A [18, 44]. Mapping the effectors on lines Rika and Kombar determined that four independent effector loci were responsible for the phenomenon, with *Ptt* isolate 6A harboring *VR1* and *VR2* contributing virulence on Rika, and *Ptt* isolate 15A harboring *VK1* and *VK2* contributing virulence on Kombar with each pair of effectors being functionally redundant [18]. Whether *Ptm_QTL7* identified on the proximal end of chromosome 2 with TR250, Kombar and Chebec in this study is *VK2*, which was initially identified on the distal end of chromosome 2 in *Ptt* is yet to be determined and will require further research. Interestingly, all four virulence loci (*VR1*, *VR2*, *VK1*, and *VK2*) interact with the barley *Spt1* dominant susceptibility locus on barley chromosome 6H [18, 44] and the Rika and Kombar alleles of *Spt1* confer reciprocal susceptibility to 6A and 15A, respectively. *Ptm_QTL9* appears to be a novel locus not previously identified and may be the first case of a single effector providing reciprocal virulence profiles shown on barley lines MXB468 and CI9819 (Fig. 6). Currently, this locus appears to parallel the same phenomena found in the host with the *Rpt5/Spt1* locus having been identified as a susceptibility locus to different isolates in Kombar and Rika and a resistant locus in CI5791 [18, 44–46]. Further research is required to determine if a single allelic gene, or multiple genes at the *Ptm_QTL9* locus may be responsible for the reciprocal virulence profiles found most strikingly with barley lines MXB468 and CI9819.

MTAs were not detected with seven of the barley lines when challenged with the US *Ptm* population. This is not surprising given that these barley lines had moderate to low differential power for the phenotypic reactions using the 1–5 *Ptm* scale [37]. This may raise the prospect of using a microscopic phenotyping scale to dissect the minute details of infection, such as that used in barley stripe rust to increase the differential power of macroscopic phenotyping [47]. In addition, failure to identify virulence/avirulence MTA on some barley lines could be due to insufficient marker saturation. Due to the fact that LD decay was estimated to be 7 kbp at an *R*^*2*^ value of 0.1 (Supplemental Fig. 2), additional MTA could have been identified with greater marker saturation as RAD-GBS is known to have intrinsic bias on SNP distribution due to the location of restriction sites and requirement to size select fragments for sequencing. This is supported by the recent *Ptm* genome assemblies showing genomes ranging from 38.2 to 42.7 Mbp in size [48] for an average marker coverage of a SNP every ~ 8 kbp across the *Ptm* genome which is greater than the estimated LD decay in this study.

Four of the top five MTAs in respect to phenotypic variation were on chromosome 3 (Table 3). These findings demonstrate the complexity of molecular interactions on *Ptm* chromosome 3, suggesting that chromosome 3 contains a reservoir of *Ptm* effectors involved in host–pathogen interactions. These findings are similar to those of the first *Ptt* GWAS that found chromosome 3 and 5 to be substantial reservoirs of effector loci [20]. Since *Ptt* and *Ptm* are not documented to have accessory chromosomes [48–50], unlike other plant pathogens such as *Fusarium oxysporium, Magnoporthe orzyae,* and *Parastagonospora nodorum* [51–53], chromosome 3 of *P. teres* may be an accessory-like region embedded within the core genome [54]. Both association and bi-parental mapping will be vital to monitor novel virulence effectors that emerge and understand how they are influenced by selection pressures such as barley variety and weather; and to determine the virulence profiles of isolates for intelligent deployment of resistant sources. Validation and characterization of the genes underlying these loci will expedite the release of resistant lines by providing the ability to test single gene interactions. In addition, diagnostic assays can be developed for determining the effectors present in pathogen populations. Lastly, the virulence effectors present in *Ptm* could be valuable in understanding how it is evolving to become a wheat pathogen.

## Conclusions

Understanding virulence loci present in pathogen populations and how they interact with the host is important for deploying effective resistance in the field. The quantity of loci detected in this study show that the barley-*Ptm* interactions have evolved to be highly complex. The power of BLINK is evident by the ability to identify a single gene underlying some of the QTL in this study. Using multiple barley lines, we also corroborate that chromosome 3 is a reservoir of *P. teres* virulence genes with the identification of seven unique loci. In addition, the identification of reciprocal virulence loci, *Ptm_QTL9* draws parallels to the *Rpt5/Spt1* locus on the host side where it has not been confirmed whether multiple genes are at play or a single allelic gene is responsible for dominant susceptibility -vs- dominant resistant responses. To our knowledge, this reciprocal virulence at a single locus is the first such case reported in a plant pathogen.

## Methods

### Isolate collection, phenotyping, and genotyping

A population of 177 viable isolates were collected from six locations representing three geographically diverse regions across ND, eastern MT, and eastern ID during the 2012 and 2013 growing seasons (Table 1). The ND isolates were collected from North Dakota State University Research and Extension Centers at Dickinson, Fargo, Langdon, and Nesson Valley. The MT population was obtained from Anheuser-Busch research plots in Sidney, MT. The ID population was collected from a commercial field approximately 32 km west of Blackfoot, ID. All sites were sampled at one time period during the growing season, with sampling at ND and MT targeting the top three leaves (flag, flag-minus-one, flag-minus-two) at full head emergence to soft dough stage. At Dickinson, Langdon, Nesson Valley, ND and Sydney, MT trap plots of the six-rowed barley variety Tradition and two-rowed barley variety Pinnacle were sampled. Only one field of Pinnacle was sampled in Fargo, ND. The sample collection from Blackfoot, ID, was isolated from 21 arbitrarily selected leaves with spot-type lesions that were collected from the upper canopy of a field of the two-rowed barley variety Moravian 69 provided by Dr. Juliet Marshall (University of Idaho).

To induce sporulation from symptomatic leaf tissue 2–3 cm leaf sections containing spot-type lesions were surface-sanitized in a 1% sodium hypochlorite solution for two minutes, then rinsed three times in sterile reverse-osmosis water and blotted dry with sterile paper towels. The leaf sections were placed on water agar plates and incubated in the dark for one to seven days. Spores consistent with *P. teres* morphology that formed along the margins of the lesions were transferred to petri plates containing V8-PDA growth medium. The V8-PDA growth media contained 150 mL of V8 juice, 10 g potato dextrose agar (Difco Laboratories Inc, Franklin Lakes, NJ, USA), 3 g calcium carbonate, and 10 g agar per liter. Single spore germination and sporulation was induced under complete dark for up to seven days. Single-spore isolation was performed a second time to ensure the isolates were monoconidial and were allowed to grow for seven to ten days on fresh V8-PDA plates in the dark, then cut into 4-mm plugs and air-dried before long term storage at -20C.

Each of the 177 *Ptm* isolates were inoculated onto a set of thirty barley lines selected for their differential response to a diverse global collection of *Ptm* isolates (Supplemental Table 1). The inoculations and disease assays were performed as described in Neupane et al. (2015) using a 1 to 5 scale with at least three independent replications [37]. In brief two to three seeds of each barley line were planted per cone, with a mean phenotypic score of two cones for each line accounting for one replication. The mean of the three replications were used as the input for association mapping. Phenotypes were also converted to a binary code to denote resistance (0) or susceptibility (1) using the the value of 3–5 as susceptible and 1–2 as resistant [37]. DNA was extracted and isolates were genotyped using a two-enzyme RAD-GBS approach based on the method of Leboldus et al. (2015) with minor modifications [22]. In brief, DNA was extracted using a modified CTAB method and RAD-GBS libraries were constructed by normalizing extracted DNA to 400 to 600 ng. The gDNA was serially digesting with the CpG methylation sensitive restriction enzymes *Hha*I and *Ape*KI, and ligated with universal and unique adapters that allowed bulking of samples and sequencing on the Ion Torrent PGM system to generate raw sequence data. Quality of raw sequences were assessed using FastQC [55] and MultiQC [56] and subsequently trimmed using Trimmomatic 0.39 [57]. Trimmed sequencing reads were aligned to the *Ptm* isolate FGOB10Ptm-1 [48] genome assembly using the *mem* command in Burrows-Wheeler Aligner 0.7.17 [58], and processed with samtools view, sort and index subcommands [59]. Genotypes were called using the *HaplotypeCaller* tool from the Genome Analysis Toolkit 4.1.9.0 [60] resulting in 195,403 unique sites. Markers were filtered using vcftools 0.1.16 [61] for genotype quality (> 30), minimum depth (≥ 3), maximum alleles (2), and missing data (90%) yielding 107,696 sites across 127 individuals. Subsequently, markers and individuals were sequentially filtered for missing data at 50% and 35% missing data thresholds to maximize data retention, resulting in 4,836 sites containing informative SNPs across the twelve *Ptm* chromosomes from 103 isolates. Missing calls were imputed using Beagle 5.1 [62] using standard settings.

### Linkage disequilibrium, population structure and kinship

Due to the fact that the BLINK [34] package utilizes LD information to infer relatedness and replace the previously developed binning method of SUPER [35] and FarmCPU [36] algorithms, neither LD pruning of markers or construction of kinship matrices were performed for downstream AM. The EMMA matrix was constructed within GAPIT using the ‘kinship.algorithm = EMMA’ command to investigate population structure. However, LD was calculated for the purpose of binning MTAs into QTL post-AM with PLINK 1.90 [63] with the following settings *ld-window-R2 0, ld-window 9999* and *ld-window-kb 5292.*

Population structure was evaluated via principal components analysis (PCA) and STRUCTURE (Q) analysis using GAPIT 3 [64] in *R 3.6.3* (R Core Team) and STRUCTURE 2.3.4 [65], respectively*.* For PCA, the generated eigenvectors for each of the principal component were used to explain at least 25% (PC4) and 50% (PC15) of the variation. STRUCTURE analysis was performed using an admixture ancestry model with a burn-in of 10,000, followed by 25,000 Monte Carlo Markov Chain replications for *k* = *1* through *k* = 10 with ten iterations to determine the optimal number of subpopulations. The resulting files were zipped and uploaded to STRUCTURE HARVESTER [66] to identify the optimal *k* value of subpopulations using the *Δk* method [67]. The optimal *k* = *2* value was used to run a new STRUCTURE analysis using a burn-in of 100,000 and 250,000 replications.

### Association mapping analyses

All association analyses were conducted in GAPIT 3 [64] using the BLINK [34] algorithm in comparison to naïve models using a general linear model. The BLINK models were tested with and without population structure that was generated via PCA and in standard or binary phenotyping formats. Each of the thirty barley lines were analyzed with the eight models separately, and the best model was selected based on an acceptable MSD value [68] and visual inspection of the QQ plot for best fit to the expected *p-*values. Any model with an MSD value less than 0.005 was considered acceptable based on the MAT type control models successfully identifying the locus to a very high confidence and with the highest MSD value of 0.004486. Bonferroni correction was used at the α-level of 0.05 and 0.01 across the 4,836 markers and MTA were considered significant at *p-*value ≤ 0.00001033912 and 0.00000206782, corresponding to -log10(*p*-value) ≥ 4.99 and 5.68, respectively. The *p*-values of models with significant MTAs were parsed out and final Manhattan and QQ plots generated with the CMplot package [39] in *R 3.6.3*. Marker effects and *R*^2^ values were calculated using single marker linear regression in *R 3.6.3*.

A candidate gene list for each interval was generated by using the non-significant flanking markers of each QTL and subsequently parsing and translating the coding sequence of each gene using the FGOB10Ptm-1 genome [48] in Geneious Prime® 2020.0.1 (https://www.geneious.com). Resulting translated proteins were examined with SignalP 2.0 [69] and EffectorP 3.0 [70]. If the translated protein was not predicted to be an effector, the protein was tested for homology to other proteins/domains using BLASTP 2.12.0 + [71, 72].

## Supplementary Information

**Additional file 1:** **Supplemental File 1. ***R*^2^valuesfor all pairwise comparisons across the chromosomes within the genome.

**Additional file 2:** **Supplemental File 2. **Translated candidategene listed used for entry into SignalP, EffectorP and BLASTP.

**Additional file 3:** **Supplemental Table 1. **Characteristic of thebarley lines used in the phenotypic assays.** Supplemental Table 2. **Mean square deviation values for all modelsperformed on each barley genotype.** Supplemental Table 3. **Characteristics of thecandidate genes underlying the 30 unique loci.

**Additional file 4:** **Supplemental Figure 1.** Principal component analysis scatter plot ofthe first two principal components color coded by isolate origin location.

**Additional file 5:** **Supplemental Figure 2.** Scree plot of the eigenvalues of the first 15principal components accounting for 50% of the variation.

**Additional file 6:** **Supplemental Figure 3.** Proportion of genetic makeup of an isolateattributed to either A. twosubpopulations (*Δk*=2) or B. sixsubpopulations (*Δk* =6) of the US *Pyrenophorateres *f. *maculata *collectionbased on 4,836 SNP markers. Grouping numbers on the *x *axis represent the location of origin with Fargo (1), Langdon(2), Dickinson (3), Nesson Valley (4), Sidney (5) and Blackfoot (6). Clusteringwas performed using STRUCTURE v2.3.4. C. Line graph constructed usingSTRUCTURE HARVESTER with Δ*k *and number of subpopulations present withinthe *Pyrenophora teres *f. *maculata *population using the STRUCTUREanalysis. The peak indicates the predicted number of subpopulations.

**Additional file 7:** **Supplemental Figure 4.** Pairwise linkage decay between allintrachromosomal marker pairs along all 12 *Pyrenophorateres *f. *maculata* chromosomes and group based on *R*^2^value, with red pixelsindicating higher linkage disequilibrium. Chromosome base position on both thex and y axis correspond to the longest *Pyrenophorateres *f. *maculata *chromosome.

**Additional file 8:** **Supplemental Figure 5.** Linkage decay plot with *R*^2^values plotted against marker physical distance alongthe respective chromosome and scaled to the largest chromosome. The blue curveis based on a nonlinear locally estimated scatterplot smoothing (loess)regression with 95% confidence interval.

**Additional file 9:** **Supplemental Figure 6.** A.Manhattan plot for mating type locus. Bonferroni correction threshold isindicated by the solid (α-level0.05) and dashed (α-level0.01) red lines. SNP density is indicated along the bottom of the plot with thecorresponding heat scale shown to the left and the 12 *Pyrenophora teres*f. *maculata *chromosomes (Chr) designated below. The mating type (MAT)locus is shown below each chromosome. B.QQ plots for corresponding models identifying the MAT type locus with 95%confidence interval shown by the shaded color. The Manhattan and QQ plots weregenerated using CMplot [39] in *R 3.6.3*.

## Data Availability

The raw sequencing data generated was submitted to the NCBI Sequence Read Archive database and is openly available under BioProject ID PRJNA749305 (https://www.ncbi.nlm.nih.gov/bioproject/PRJNA749305) and accession numbers SAMN20363786 through SAMN20363888.

## Abbreviations

AM: Association mapping
BLINK: Bayesian-information and Linkage-disequilibrium Iteratively Nested Keyway
ID: Idaho
LD: Linkage Disequilibrium
MAT: Mating type
MSD: Mean Square Difference
MT: Montana
MTA: Marker Trait Association
ND: North Dakota
PCA: Principal Component Analysis
*Ptm*: *Pyrenophora teres* F. *maculata*
*Ptt*: *Pyrenophora teres* F. *teres*
QTL: Quantitative Trait Loci
RAD-GBS: Restriction site Associated DNA – Genotyping by Sequencing
SFNB: Spot Form Net Blotch
SNP: Single Nucleotide Polymorphism
