# Supplementary material for: Association mapping reveals a reciprocal virulence/avirulence locus within diverse US Pyrenophora teres f. maculata isolates
Source: BMC Genomics. 2022 Apr 9;23:285. doi: 10.1186/s12864-022-08529-1 (PMC8994276; doi:10.1186/s12864-022-08529-1)
Supplement: Supplementary file 3 — Additional file 3: Supplemental Table 1. Characteristic of thebarley lines used in the phenotypic assays. Supplemental Table 2. Mean square deviation values for all modelsperformed on each barley genotype. Supplemental Table 3. Characteristics of thecandidate genes underlying the 30 unique loci. [file 12864_2022_8529_MOESM3_ESM.docx]

**Supplemental Table 1.** Characteristic of the barley lines used in the phenotypic assays.

| Barley Genotype | Alternative Names | Country of Origin | Row Type | Accession Type | Reference |
| --- | --- | --- | --- | --- | --- |
| Pinnacle | PI 643354 | United States | Two | Cultivar |  |
| 81-82/033 |  |  |  | Cultivar | McLean et al 2012 |
| Arimont | CIho 15509 | United States | Six | Cultivar | Karki & Sharp 1986; McLean et al. 2012, 2014 |
| Chebec | PI 606292 | Australia | Two | Cultivar | McLean et al. 2012, 2014 |
| Keel |  | Australia | Two | Cultivar | Williams et al 2003; McLean et al 2010, 2012, 2014 |
| Kombar | CIho 15694 | United States | Six | Cultivar | McLean et al 2010, 2012, 2014 |
| Skiff | PI 573727 | Australia | Two | Cultivar | Wu et al 2003; McLean et al 2010, 2012, 2014 |
| CI3576 | Sidi, 77a | Egypt | Two | Landrace |  |
| CI5791 | PI95095, CIho 5791 | Ethiopia | Two | Landrace | Karki & Sharp 1986; Arabi et al 1992;  McLean et al 2012, 2014; Wu et al 2003 |
| CI7584 | Tenn. Awnless D22-5 | United States | Six | Breeding Line | Karki & Sharp 1986; McLean et al 2010; Wu et al 2003 |
| CI9214 | PI 186125, Chae-Rae-Baec | South Korea | Six | Landrace | Karki & Sharp 1986; Williams et al 2003;  McLean et al 2010, 2012, 2014 |
| CI9776 | PI 195896, Rabat 071, CIho 9776 | Morocco | Six | Cultivar | Karki & Sharp 1986; McLean et al 2012 |
| CI9819 | PI 195985, 9839, CIho 9819 | Ethiopia | Two | Landrace | Karki & Sharp 1986; Wu et al 2003 |
| CIho14219 | 4042, CI 14219 | Mongolia | Two | Landrace | Neupane et al. 2015 |
| CIho2353 | Moiris | Turkmenistan | Six | Unknown | Neupane et al. 2015 |
| CIho3694 | 90b | Egypt | Two | Landrace | Neupane et al. 2015 |
| CIho4050 | 4021 | Mongolia | Six | Landrace | Neupane et al. 2015 |
| MXB468 |  | (CIMMYT) |  | Landrace | McLean et al 2012 |
| PI269151 | Fresa, CIho 11418 | United Kingdom | Two | Cultivar |  |
| PI369731 | Medicum 8955 | Kazakstan | Two | Cultivar |  |
| PI392501 | Welgevallen 65-31-36 | South Africa | Two | Breeding Line |  |
| PI467375 | Brehat | France | Two | Landrace |  |
| PI467729 | Moyjar | Norway | Two | Cultivar |  |
| PI485524 | Cerise | United Kingdom | Two | Cultivar |  |
| PI498434 | Kuaka | New Zealand | Two | Cultivar |  |
| PI513205 | 1232/1, 30315, Urbush | Pakistan | Six | Landrace |  |
| PI565826 | Liao Tang Zai Lai, ZDM 1350 | China | Six | Cultivar | Neupane et al. 2015 |
| PI573662 | S-26, HOR 9611, NSGC 345 | Georgia | Two | Landrace | Neupane et al. 2015 |
| TR250 |  | Canada |  |  | McLean et al 2012, 2014 |
| TR326 |  | Canada |  |  | McLean et al 2012, 2014 |

**Supplemental Table 2.** Mean square deviation values for all models performed on each barley genotype.

| Genotype | Naïve | Naïve_Binary_ | BLINK | BLINK_Binary_ | BLINK_PC4_ | BLINK_PC4+Binary_ | BLINK_PC15_ | BLINK_PC15+Binary_ | Best Model | MTA(s) |
| --- | --- | --- | --- | --- | --- | --- | --- | --- | --- | --- |
| MAT | 0.000840 | 0.000840 | 0.000808 | 0.000808 | 0.000924 | 0.000924 | 0.004486 | 0.004486 | BLINK_PC4_ | 1 |
| Pinnacle | 0.006516 | 0.008949 | 0.000517 | 0.329062 | 0.000165 | 0.324066 | 0.000025 | 0.319535 | BLINK_PC4_ | None |
| Ciho14219 | 0.000591 | NA | 0.000973 | NA | 0.000534 | NA | 0.000594 | NA | BLINK_PC15_ | None |
| 81-82/033 | 0.003972 | 0.000631 | 0.000053 | 0.000557 | 0.000435 | 0.000057 | 0.000027 | 0.000429 | BLINK_PC4+Binary_ | None |
| Arimont | 0.009096 | 0.011778 | 0.000166 | 0.001487 | 0.001424 | 0.000252 | 0.001668 | 0.000260 | BLINK_PC4+Binary_ | None |
| Chebec | 0.010144 | 0.010665 | 0.000241 | 0.004170 | 0.005011 | 0.019598 | 0.000052 | 0.000138 | BLINK_PC15+Binary_ | 4 |
| Keel | 0.007404 | 0.010221 | 0.000768 | 0.000265 | 0.000049 | 0.000017 | 0.000068 | 0.000053 | BLINK_PC4_ | None |
| Kombar | 0.028361 | 0.020499 | 0.001132 | 0.000266 | 0.001120 | 0.000781 | 0.000029 | 0.000237 | BLINK_Binary_ | 4 |
| Skiff | 0.022649 | 0.010478 | 0.000314 | 0.080741 | 0.000003 | 0.069628 | 0.000217 | 0.016545 | BLINK_PC4_ | None |
| CI3576 | 0.040497 | 0.023403 | 0.000021 | 0.000379 | 0.002159 | 0.000385 | 0.000041 | 0.000022 | BLINK | 2 |
| CI5791 | 0.000514 | 0.008141 | 0.000095 | 0.011299 | 0.000152 | 0.002942 | 0.000157 | 0.000896 | BLINK_PC15+Binary_ | 1 |
| CI7584 | 0.020443 | 0.021578 | 0.000479 | 0.000427 | 0.000223 | 0.000095 | 0.000221 | 0.000410 | BLINK_Binary_ | 1 |
| CI9214 | 0.001972 | 0.012403 | 0.000269 | 0.013600 | 0.000172 | 0.003040 | 0.000207 | 0.125914 | BLINK | 2 |
| CI9776 | 0.022805 | 0.017011 | 0.000058 | 0.000083 | 0.000074 | 0.000033 | 0.000079 | 0.000279 | BLINK | 1 |
| CI9819 | 0.034517 | 0.026783 | 0.000235 | 0.000362 | 0.000945 | 0.001747 | 0.000059 | 0.000336 | BLINK_Binary_ | 1 |
| CIho2353 | 0.014734 | 0.005353 | 0.001585 | 0.034878 | 0.000027 | 0.024515 | 0.000020 | 0.002816 | BLINK_PC15_ | None |
| CIho3694 | 0.035276 | 0.031370 | 0.001449 | 0.010735 | 0.000021 | 0.000053 | 0.000115 | 0.000192 | BLINK | 2 |
| Ciho4050 | 0.002400 | 0.030280 | 0.000558 | 0.044504 | 0.000336 | 0.002489 | 0.000287 | NA | BLINK_PC15_ | 3 |
| MXB468 | 0.058531 | 0.058131 | 0.005061 | 0.002933 | 0.000954 | 0.000122 | 0.004486 | 0.000162 | BLINK_PC4_ | 1 |
| PI269151 | 0.021849 | 0.005353 | 0.000041 | 0.010440 | 0.000077 | 0.000216 | 0.000675 | 0.000257 | BLINK_PC4+Binary_ | 1 |
| PI369731 | 0.034940 | 0.028628 | 0.000093 | 0.003138 | 0.000167 | 0.000022 | 0.000054 | 0.000029 | BLINK | 1 |
| PI392501 | 0.005488 | 0.003776 | 0.000026 | 0.008139 | 0.000038 | 0.000082 | 0.000108 | 0.000399 | BLINK_PC4_ | None |
| PI467375 | 0.020060 | 0.007884 | 0.000100 | 0.000182 | 0.000044 | 0.000039 | 0.000001 | 0.000012 | BLINK_Binary_ | 1 |
| PI467729 | 0.011113 | 0.003774 | 0.001427 | 0.005586 | 0.000126 | 0.000053 | 0.000243 | 0.000319 | BLINK | 2 |
| PI485524 | 0.004173 | 0.001728 | 0.000112 | 0.002641 | 0.000032 | 0.000293 | 0.000199 | 0.000029 | BLINK_PC4+Binary_ | None |
| PI498434 | 0.027379 | 0.009731 | 0.002711 | 0.007091 | 0.000377 | 0.000102 | 0.000507 | 0.000164 | BLINK_PC4+Binary_ | 2 |
| PI513205 | 0.291868 | 0.001485 | 0.000329 | 0.000560 | 0.000135 | 0.000198 | 0.000152 | 0.000109 | BLINK_PC4_ | None |
| PI565826 | 0.019407 | 0.015571 | 0.004230 | 0.000129 | 0.000088 | 0.000025 | 0.000035 | 0.000115 | BLINK_PC15_ | 1 |
| PI573662 | 0.011727 | 0.001483 | 0.006137 | 0.000338 | 0.000366 | 0.000030 | 0.001402 | 0.001097 | BLINK | 2 |
| TR250 | 0.010825 | 0.013110 | 0.004248 | 0.000220 | 0.000026 | 0.000020 | 0.000183 | 0.000420 | BLINK | 3 |
| TR326 | 0.011270 | 0.000872 | 0.000064 | 0.001153 | 0.000209 | 0.000306 | 0.001600 | 0.004422 | BLINK_PC4+Binary_ | 2 |

**Supplemental Table 3.** Characteristics of the candidate genes underlying the 30 unique loci.

|  |  |  |  | EffectorP 3.0^1^ | | |  |
| --- | --- | --- | --- | --- | --- | --- | --- |
| Loci | Interval/kp | Candidate Gene(s) | SignalP 5.0 | Cytoplasmic | Apoplastic | Non^2^ | BLAST^2^ |
| *Ptm_QTL1* | 7.6 | 2 | 0 | 0 | 1 | 1 | Trichoplein |
| *Ptm_QTL2* | 4.5 | 3 | 0 | 1 | 1 | 2 | Ankyrin repeat protein  Integral membrane protein |
| *Ptm_QTL3* | 4.7 | 3 | 0 | 0 | 0 | 3 | Hypothetical  Hypothetical  Hydrolase |
| *Ptm_QTL4* | 108.7 | 16 | 0 | 11 | 2 | 4 | Tetratricopeptide repeat protein  Transferase  Major facilitator superfamily protein Hypothetical |
| *Ptm_QTL5* | 98.8 | 16 | 1 | 11 | 0 | 5 | Dimer-Tnp-hAT domain protein  Hypothetical  Hypothetical  YjeF domain protein  Serine/threonine protein phosphatase |
| *Ptm_QTL6* | 20.0 | 4 | 0 | 1 | 0 | 3 | Sulfate permease  Hypothetical  Vacuolar protein sorting-associated protein |
| *Ptm_QTL7* | 7.2 | 3 | 0 | 0 | 0 | 3 | Acyltransferase  Sugar kinase  Rit1 C multidomain protein |
| *Ptm_QTL8* | 38.4 | 16 | 4 | 5 | 2 | 10 | Nucleosome binding factor  Serine carboxypeptidase  Hypothetical  Hypothetical  Glutaminase  Hypothetical  Methyltransferse  Cryptochrome/photolyase protein  Glycosyltransferase  Cytochrome C oxidase protein |
| *Ptm_QTL9* | 35.3 | 10 | 1 | 7 | 0 | 3 | Nonribosomal peptide synthetase  Hypothetical  Cytochrome P450 |
| *Ptm_QTL10* | 95.3 | 23 | 2 | 12 | 0 | 11 | Nonribosomal peptide synthetase  None  Nonribosomal peptide synthetase  None  Dimer-Tnp-hAT domain protein  Threonine-rich GPI-anchored glycoprotein  Hypothetical  SH3 domain protein  Transmembrane receptor  Cytoplasmic tRNA 2-thiolation protein  RNA helicase A |
| *Ptm_QTL11* | 34.9 | 15 | 1 | 6 | 1 | 8 | Hypothetical  Hypothetical  Rph RNase PH  Ubiquitin-protein ligase  Hypothetical  Importin subunit alpha  Hypothetical  Hypothetical |
| *Ptm_QTL12* | 0.06 | 1 | 0 | 0 | 0 | 1 | Hexotransporter |
| *Ptm_QTL13* | 20.5 | 4 | 0 | 0 | 0 | 4 | Methyltransferase  Beta-glucosidase  DUF726 protein  Hypothetical |
| *Ptm_QTL14* | 53.7 | 15 | 1 | 6 | 0 | 9 | Hypothetical  Hypothetical  None  Lysophospholipase  Dynamin N domain protein  Glycosyltransferase  Tyrosine protein phosphatase  Acetyltransferase  ISWI chromatin-remodelling complex ATPase |
| *Ptm_QTL15* | 8.5 | 5 | 2 | 0 | 2 | 3 | Peroxidase-2 domain protein  None  Ribose-phosphate pyrophosphokinase |
| *Ptm_QTL16* | 8.0 | 3 | 0 | 2 | 0 | 1 | SacI domain protein |
| *Ptm_QTL17* | 14.9 | 3 | 0 | 0 | 0 | 3 | Phosphoinositide phospholipase C  Hypothetical  Phosphatidyl synthase |
| *Ptm_QTL18* | 24.5 | 9 | 0 | 2 | 0 | 7 | Ankyrin repeat protein  Hypothetical  Cytochrome P450  Fatty acid synthase  Cytochrome P450  Cytochrome P450  Glutamate-cysteine ligase |
| *Ptm_QTL19* | 0.09 | 1 | 0 | 0 | 0 | 1 | Eisosome |
| *Ptm_QTL20* | 21.7 | 5 | 0 | 0 | 0 | 5 | Hypothetical  Hypothetical  Ring finger protein  Hypothetical  Hypothetical |
| *Ptm_QTL21* | 36.4 | 14 | 1 | 5 | 1 | 8 | Zing finger domain-containing protein  Hypothetical  Major facilitator superfamily protein  GTPase-activating protein  Condensin complex protein  Sodium/calcium antiporter  Aromatic-ring hydroxylase  Toxin biosynthesis regulatory protein |
| *Ptm_QTL22* | 28.1 | 5 | 0 | 2 | 0 | 3 | Ubiquitin-protein ligase  Major facilitator superfamily protein  Zinc finger domain-containing protein |
| *Ptm_QTL23* | 3.5 | 1 | 0 | 1 | 0 | 0 |  |
| *Ptm_QTL24* | 9.4 | 3 | 0 | 0 | 0 | 3 | GTP-binding protein  Phosphohydrolase  Hypothetical |
| *Ptm_QTL25* | 18.0 | 6 | 0 | 0 | 0 | 6 | Hypothetical  Hydrolase  GAL4 domain-containing protein  Hypothetical  Hypothetical  Dehydrogenase |
| *Ptm_QTL26* | 0.08 | 2 | 0 | 1 | 0 | 1 | Kinase |
| *Ptm_QTL27* | 6.3 | 2 | 0 | 2 | 0 | 0 |  |
| *Ptm_QTL28* | 15.3 | 7 | 0 | 1 | 0 | 6 | FAD dependent oxidoreductase  None  Transmembrane transporter  Frutosyl peptide oxidase  Heat shock transcription factor  F-box multi-domain protein |
| *Ptm_QTL29* | 10.5 | 4 | 1 | 2 | 0 | 2 | Leupeptin-inactivating enzyme  Serine/threonine protein kinase |
| *Ptm_QTL30* | 0.01 | 1 | 0 | 0 | 0 | 1 | Polyketide Synthase |
| Total | 734.94 | 202 | 14 | 78 | 10 | 117 |  |

^1^May exceed total number of candidate genes if an effector is simultaneously predicted as an apoplastic and a cytoplasmic effector.

^2^Only genes predicted to be non-effectors were tested for protein homology using BLAST.
