# Supplementary figures and images for: Association mapping reveals a reciprocal virulence/avirulence locus within diverse US Pyrenophora teres f. maculata isolates
Source: BMC Genomics. 2022 Apr 9;23:285. doi: 10.1186/s12864-022-08529-1 (PMC8994276; doi:10.1186/s12864-022-08529-1)

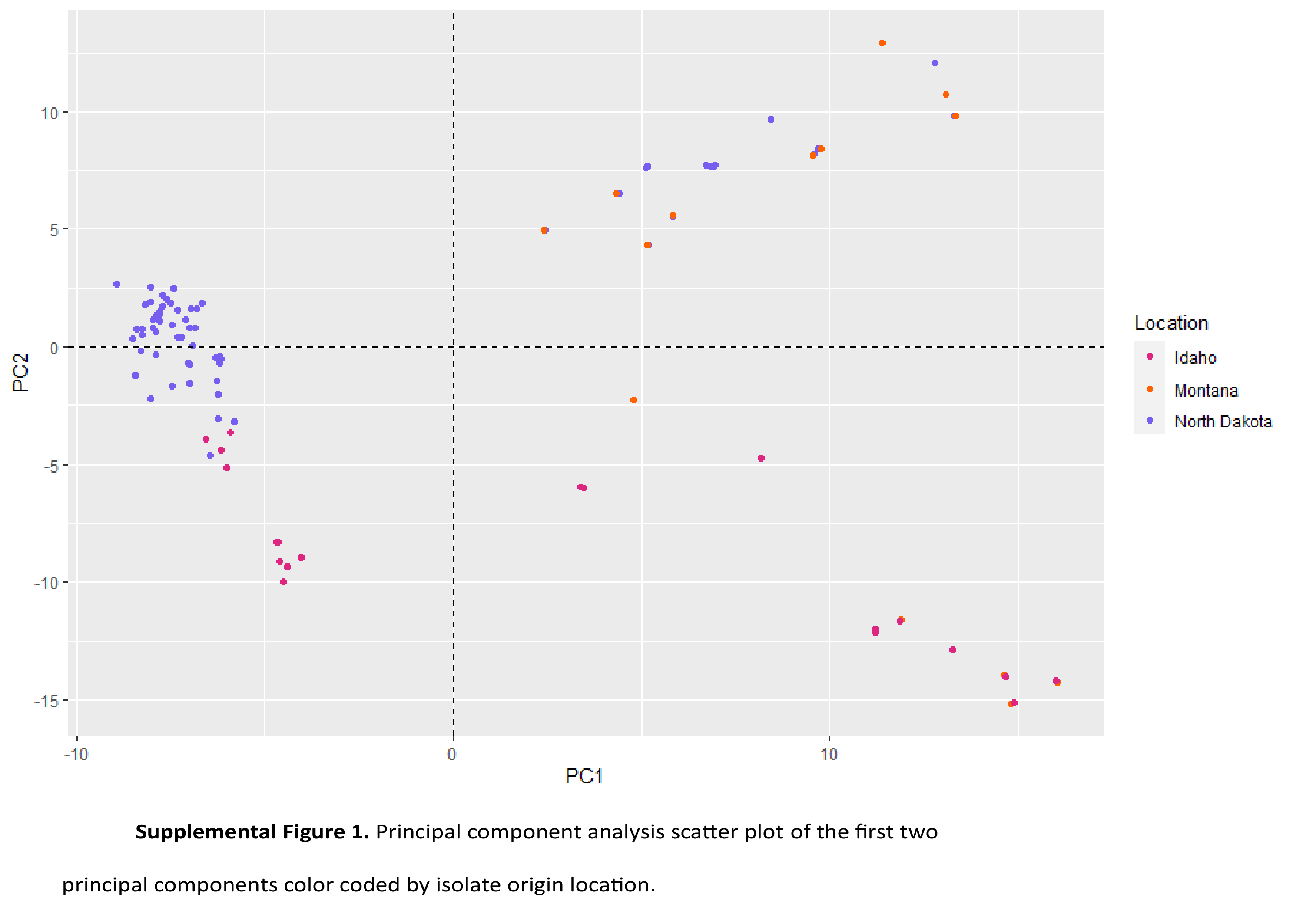

Supplement: Supplementary file 4 — Additional file 4: Supplemental Figure 1. Principal component analysis scatter plot ofthe first two principal components color coded by isolate origin location. [file 12864_2022_8529_MOESM4_ESM.png]

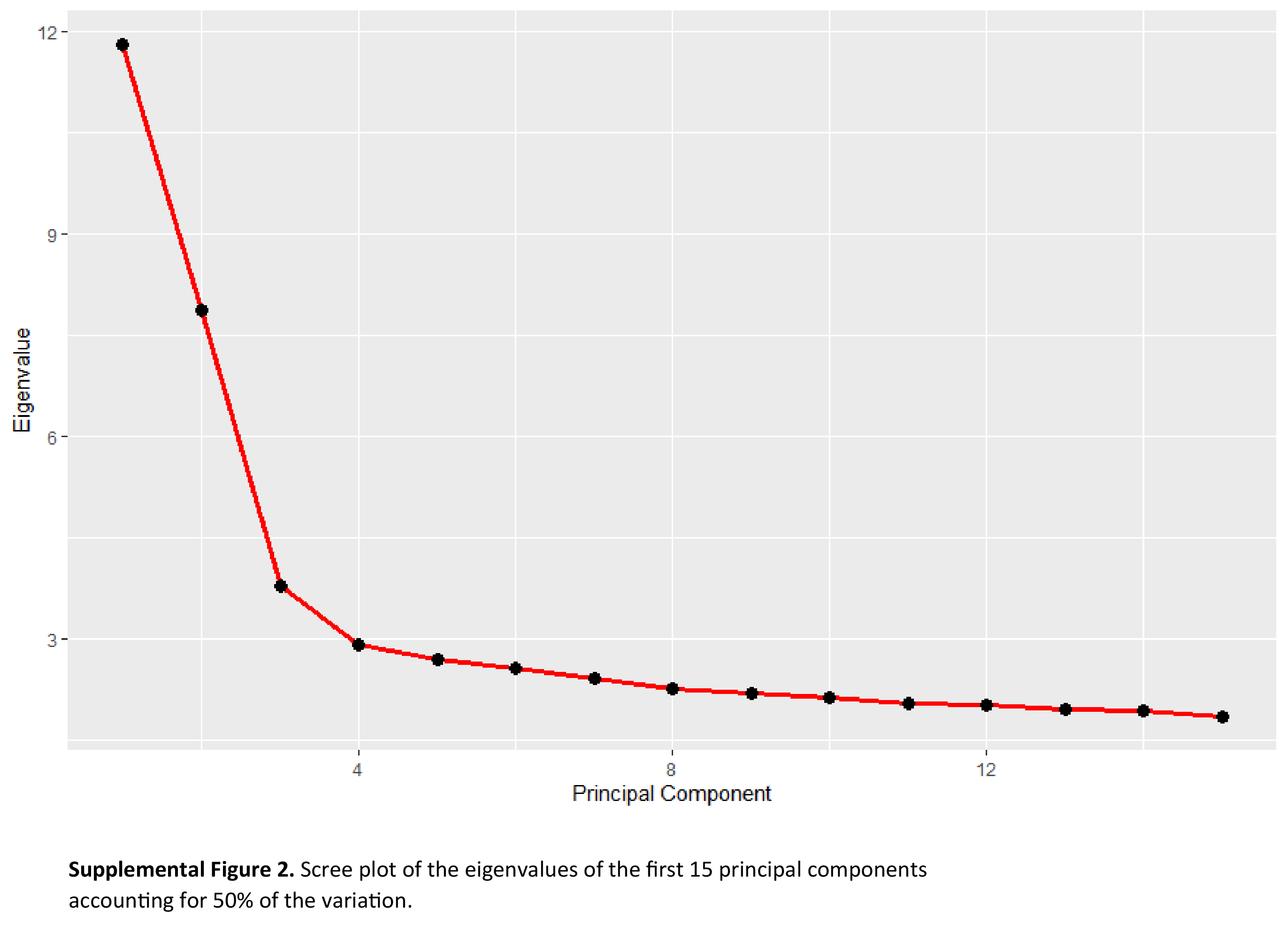

Supplement: Supplementary file 5 — Additional file 5: Supplemental Figure 2. Scree plot of the eigenvalues of the first 15principal components accounting for 50% of the variation. [file 12864_2022_8529_MOESM5_ESM.png]

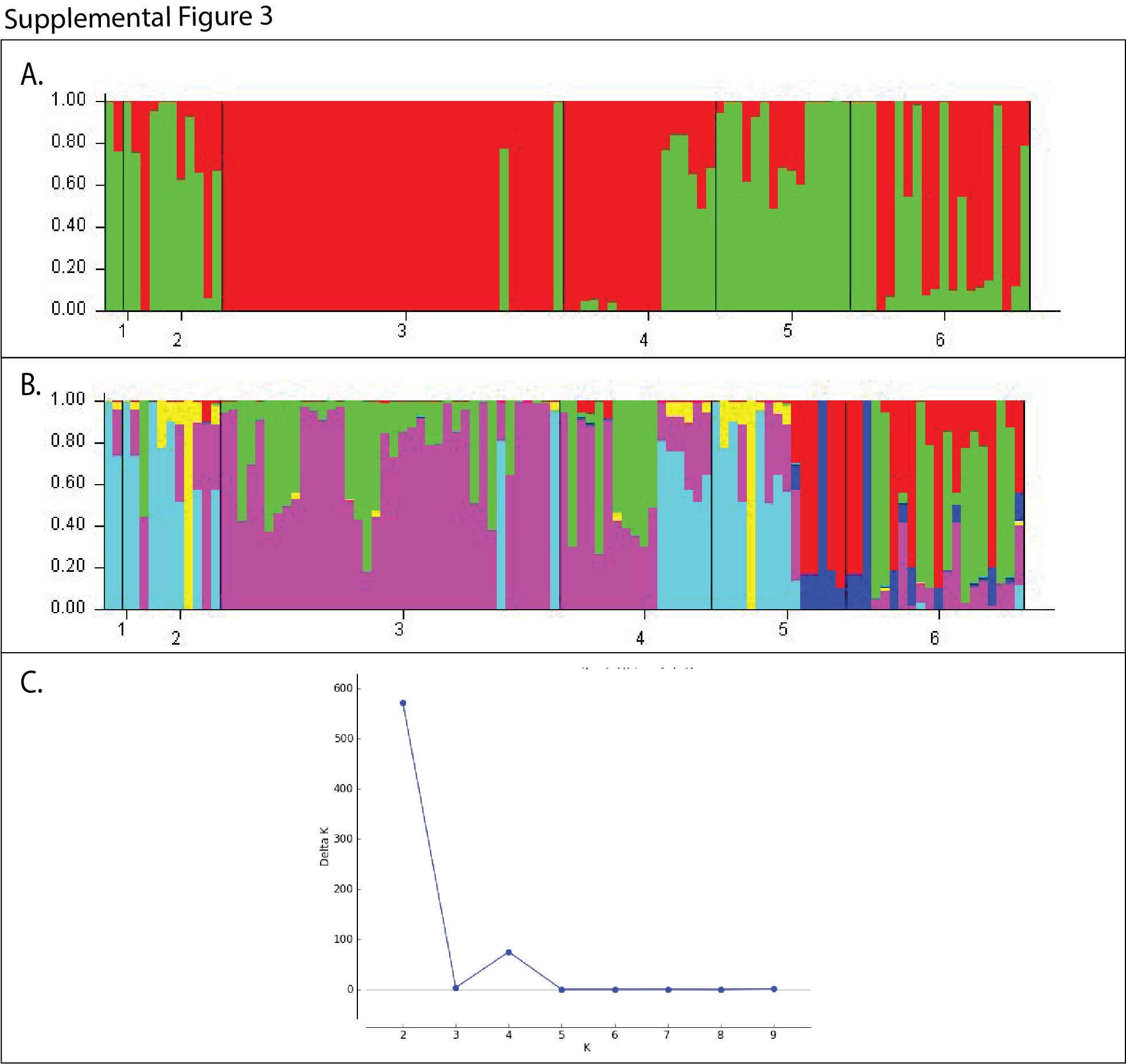

Supplement: Supplementary file 6 — Additional file 6: Supplemental Figure 3. Proportion of genetic makeup of an isolateattributed to either A. twosubpopulations (Δk=2) or B. sixsubpopulations (Δk =6) of the US Pyrenophorateres f. maculata collectionbased on 4,836 SNP markers. Grouping numbers on the x axis represent the location of origin with Fargo (1), Langdon(2), Dickinson (3), Nesson Valley (4), Sidney (5) and Blackfoot (6). Clusteringwas performed using STRUCTURE v2.3.4. C. Line graph constructed usingSTRUCTURE HARVESTER with Δk and number of subpopulations present withinthe Pyrenophora teres f. maculata population using the STRUCTUREanalysis. The peak indicates the predicted number of subpopulations. [file 12864_2022_8529_MOESM6_ESM.png]

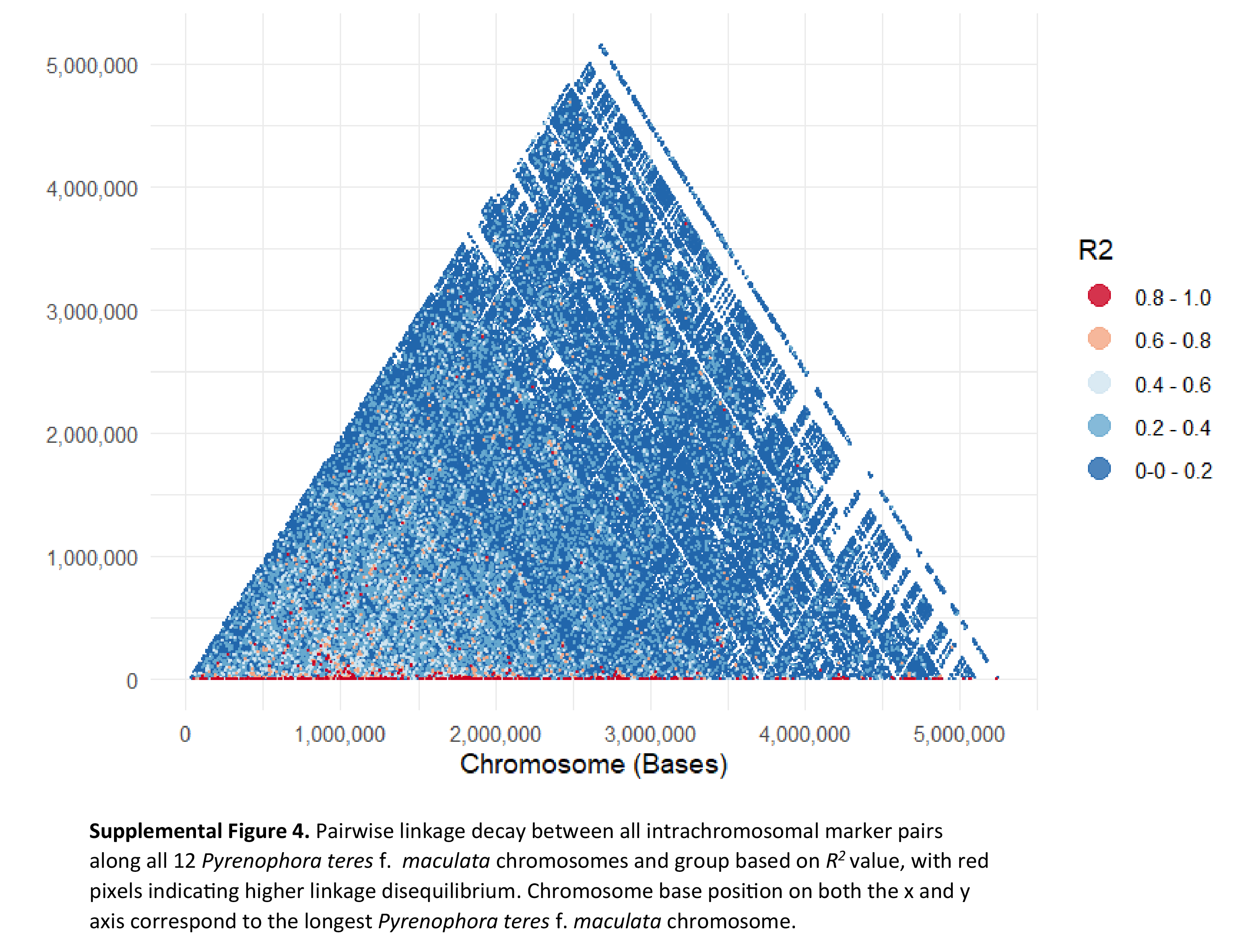

Supplement: Supplementary file 7 — Additional file 7: Supplemental Figure 4. Pairwise linkage decay between allintrachromosomal marker pairs along all 12 Pyrenophorateres f. maculata chromosomes and group based on R2value, with red pixelsindicating higher linkage disequilibrium. Chromosome base position on both thex and y axis correspond to the longest Pyrenophorateres f. maculata chromosome. [file 12864_2022_8529_MOESM7_ESM.png]

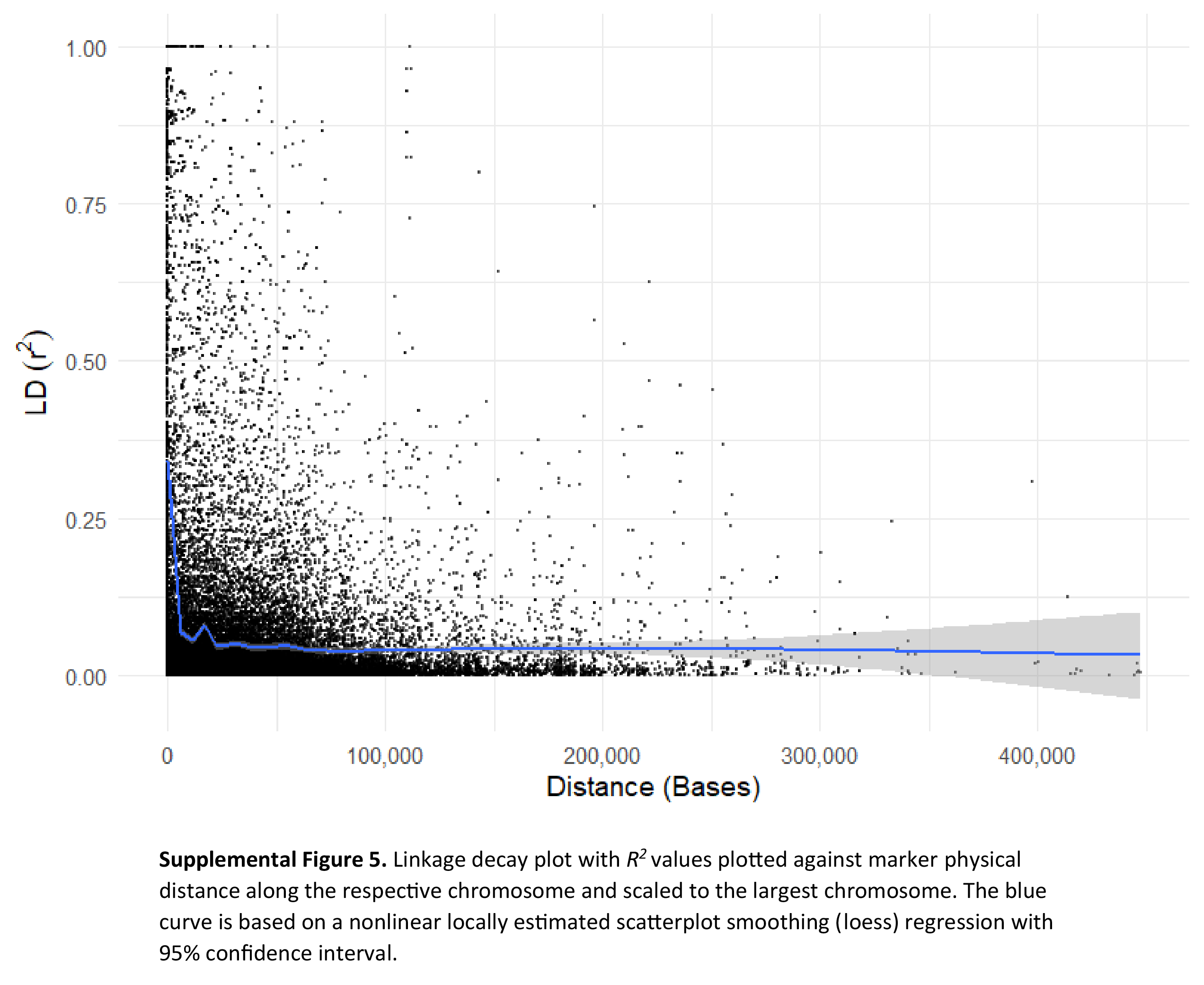

Supplement: Supplementary file 8 — Additional file 8: Supplemental Figure 5. Linkage decay plot with R2values plotted against marker physical distance alongthe respective chromosome and scaled to the largest chromosome. The blue curveis based on a nonlinear locally estimated scatterplot smoothing (loess)regression with 95% confidence interval. [file 12864_2022_8529_MOESM8_ESM.png]
