# Supplementary material for: Association mapping reveals a reciprocal virulence/avirulence locus within diverse US Pyrenophora teres f. maculata isolates
Source: BMC Genomics. 2022 Apr 9;23:285. doi: 10.1186/s12864-022-08529-1 (PMC8994276; doi:10.1186/s12864-022-08529-1)
Supplement: Supplementary file 9 — Additional file 9: Supplemental Figure 6. A.Manhattan plot for mating type locus. Bonferroni correction threshold isindicated by the solid (α-level0.05) and dashed (α-level0.01) red lines. SNP density is indicated along the bottom of the plot with thecorresponding heat scale shown to the left and the 12 Pyrenophora teresf. maculata chromosomes (Chr) designated below. The mating type (MAT)locus is shown below each chromosome. B.QQ plots for corresponding models identifying the MAT type locus with 95%confidence interval shown by the shaded color. The Manhattan and QQ plots weregenerated using CMplot [39] in R 3.6.3. [file 12864_2022_8529_MOESM9_ESM.pdf]

Supplemental Figure 6

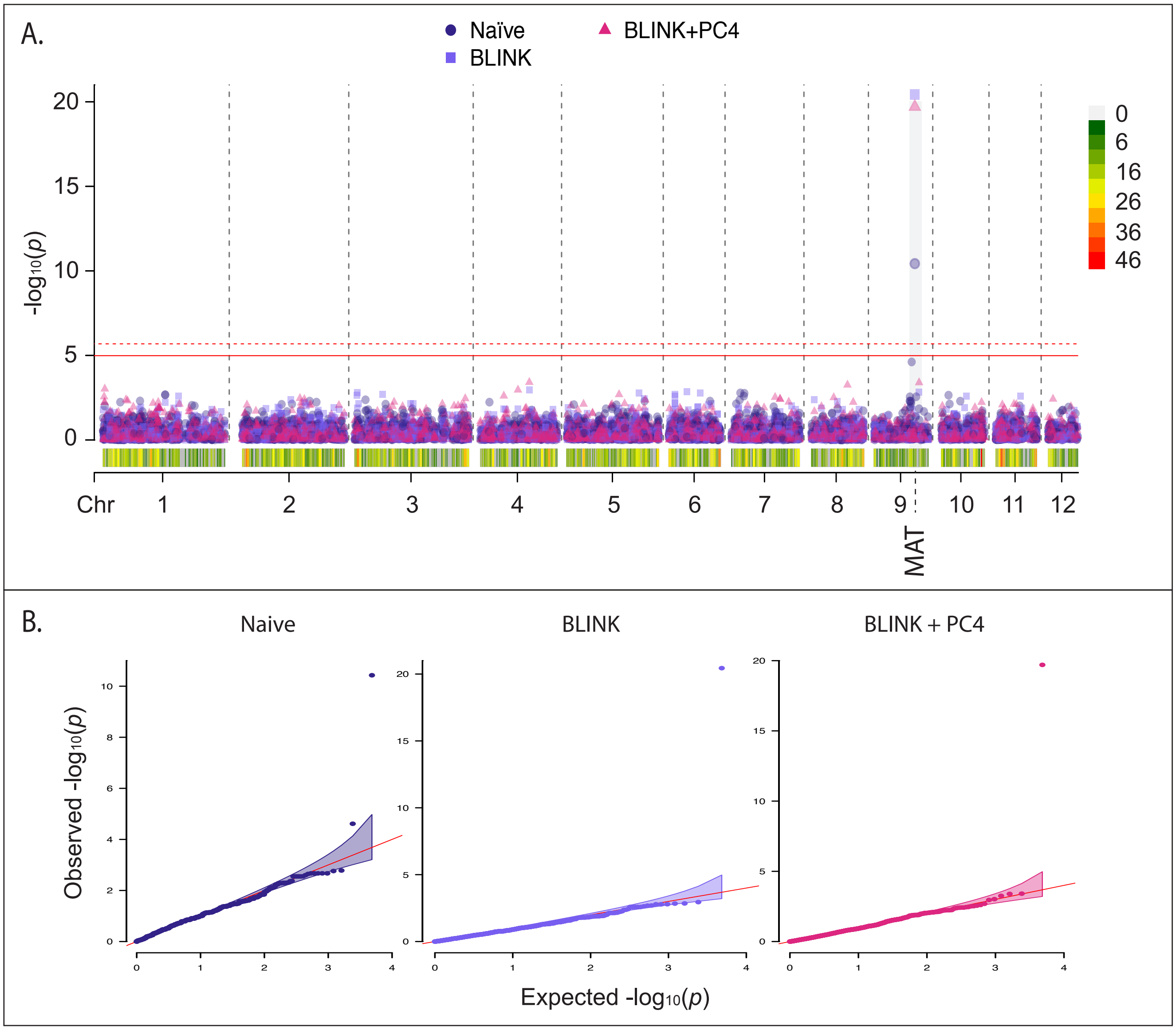

Supplemental Figure 6. A. Manhattan plot for mating type locus. Bonferroni correction threshold is indicated by the solid (α-level 0.05) and dashed (α-level 0.01) red lines. SNP density is indicated along the bottom of the plot with the corresponding heat scale shown to the left and the 12 Pyrenophora teres f. maculata chromosomes (Chr) designated below. The mating (MAT) type locus is shown below each chromosome. B. QQ plots for corresponding models identifying the MAT type locus with 95% confidence interval shown by the shaded color. The Manhattan and QQ plots were generated using CMplot [67] in R 3.6.3.
